# Genetic diversity maintained among fragmented populations of a tree undergoing range contraction

**DOI:** 10.1038/s41437-018-0132-8

**Published:** 2018-08-15

**Authors:** James S. Borrell, Nian Wang, Richard A. Nichols, Richard J. A. Buggs

**Affiliations:** 1 Jodrell Laboratory, Royal Botanic Gardens, Kew, Richmond, Surrey, TW9 3DS UK; 2 School of Biological and Chemical Sciences, Queen Mary University of London, London, E1 4NS UK; 3 Present Address: College of Forestry, Shandong Agricultural University, Tai’an city, 271018 Shandong Province China

## Abstract

Dwarf birch (*Betula nana*) has a widespread boreal distribution but has declined significantly in Britain where populations are now highly fragmented. We analyzed the genetic diversity of these fragmented populations using markers that differ in mutation rate: conventional microsatellites markers (PCR-SSRs), RADseq generated transition and transversion SNPs (RAD-SNPs), and microsatellite markers mined from RADseq reads (RAD-SSRs). We estimated the current population sizes by census and indirectly, from the linkage-disequilibrium found in the genetic surveys. The two types of estimate were highly correlated. Overall, we found genetic diversity to be only slightly lower in Britain than across a comparable area in Scandinavia where populations are large and continuous. While the ensemble of British fragments maintain diversity levels close to Scandinavian populations, individually they have drifted apart and lost diversity; particularly the smaller populations. An ABC analysis, based on coalescent models, favors demographic scenarios in which Britain maintained high levels of genetic diversity through post-glacial re-colonization. This diversity has subsequently been partitioned into population fragments that have recently lost diversity at a rate corresponding to the current population-size estimates. We conclude that the British population fragments retain sufficient genetic resources to be the basis of conservation and re-planting programmes. Use of markers with different mutation rates gives us greater confidence and insight than one marker set could have alone, and we suggest that RAD-SSRs are particularly useful as high mutation-rate marker set with a well-specified ascertainment bias, which are widely available yet often neglected in existing RAD datasets.

## Introduction

“In too small a population (1/4 *N* much greater than *u* and *s*) there is nearly complete fixation, little variation, little effect of selection and thus a static condition modified occasionally by chance fixation of new mutations leading inevitably to degeneration and extinction” (Wright 1931 p. 157) [*N* = population size, *u* = mutation rate and *s* = selection co-efficient]

Wright (1931) showed that chance will dominate in the evolution of genetic loci in a population whose effective size is low relative to the mutation rates and selection coefficients acting upon its loci. He predicted this would lead to the demise of those populations. This insight has been widely applied in conservation (Ellstrand and Elam 1993; Koskela et al. 2013), but genetic studies in plants seeking to show that population fragmentation leads to small, drifting, degenerating populations have given mixed results (Ellstrand and Elam 1993; Young et al. 1996), especially for trees (Kramer et al. 2008; Piotti 2009; Bacles and Jump 2011; Vranckx et al. 2012; Martins et al. 2016). This has led to suggestions that traits commonly found in tree species make them resilient to deleterious consequences of fragmentation (Lowe et al. 2015).

Our study species is dwarf birch (*Betula nana*), a monecious wind-pollinated, subarctic dwarf tree. *Betula* are generally considered self-incompatible, however, low levels of selfing has been reported in many species (Clausen 1966). Dwarf birch is nationally scarce in Britain where it appears to have declined significantly over the past centuries leading to fragmentation severity varying in space and time (Aston 1984; Wang et al. 2014). Threats include heavy browsing pressure, frequent moorland burning, potential genetic degradation, hybridization and climate change (Gilbert and Di Cosmo 2009; Zohren et al. 2016). The species is now under active conservation management on some Highland estates. By contrast in northern Scandinavia dwarf birch has an extensive range with largely continuous populations. The British and Scandinavian regions are thought to have a similar re-colonization history (Alsos et al. 2007; Eidesen et al. 2015) since the last glacial maximum (LGM) and so may provide a useful comparison.

Here, we quantify the decline of dwarf birch across Britain over the past three centuries, locate its extant populations and estimate their current census population sizes. We survey their genetic diversity with four genomic marker sets with a range of mutation rates and processes providing complementary information: conventional microsatellites amplified with PCR primers (for clarity termed here PCR-SSRs), single-nucleotide polymorphisms from RAD-sequencing reads divided into transitions (RAD-SNP_ti_) and transversions (RAD-SNP_tv_), and microsatellites data-mined from RAD-sequencing reads (RAD-SSRs). Typically, indel mutations generating variation at SSR loci occur more frequently than substitution mutations that cause variation of SNPs (Li et al. 2002; Payseur and Cutter 2006) and transition SNPs mutate faster than transversions (Zhang and Gerstein 2003; Van Bers et al. 2010).

Due to their mutational mechanism, SSRs are expected to have higher rates of homoplasy, which may erode signatures of population differentiation over time (Estoup et al. 2002; Coates et al. 2009). Conversely, SNPs may lack the variation to distinguish recent demographic events (Morin et al. 2004). The different marker sets may also have contrasting ascertainment biases: PCR-based SSRs in particular, are likely to be from primers chosen to amplify loci with high heterozygosity. Another difference is that null alleles are more likely to occur in SSR surveys, affecting population genetic measures (Chapuis and Estoup 2007).

We use our data to address the conservation genetic status of dwarf birch populations in Scotland and its past demography. We compare overall levels of diversity between British and Scandinavian populations, and investigate how this is partitioned among British fragments. To investigate the relative importance of recent genetic drift in generating these patterns of diversity, we estimated the current census and effective population sizes. We use ABC analysis of coalescent models to make inferences about the historical events that may have contributed to the current distribution of genetic diversity among remaining British populations. Finally, we discuss our findings in the context of conserving genetic diversity in fragmented plant populations and the use of markers with differing mutation rates in such studies.

## Materials and methods

### Plant sampling

To quantify the decline of *Betula nana* in Britain, 2245 records from the years 1777 to 2012 were collated from national databases and conservation organizations. Historical sampling effort has varied substantially, concurrent with a hypothesized decline in *B. nana*. Thus, to account for this variation we also collected 23,990 records over the same period for *B. pubescens*, a related Birch species with a broader distribution that is considerably more abundant and with no significant recent population trends (Wang et al. 2014). *Betula pubescens* observations were divided into three time periods with equal numbers of records, *B. nana* records were then binned among these time periods to a geographical resolution of 0.1 degrees, with duplicates removed, and plotted using the ggplot package (Wickham 2009) in R software (R Development Core Team 2014) and RStudio (RStudio Team 2015). *B. nana* records were also assessed in Finland although recording schemes are sparse and recent. Thus, we referred to prevalence maps developed for the Vascular Plant Atlas of Finland (Lampinen and Lahti 2016), which support observations that the species is widespread and abundant.

**Fig. 1:**
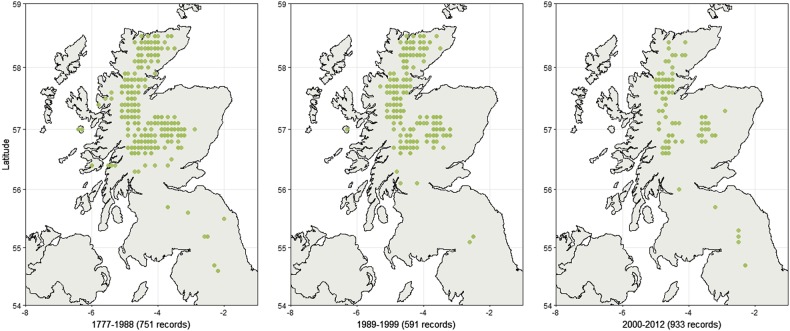
Historical records of *Betula nana* in Britain binned to 0.1 degree resolution, showing progressive range decline and fragmentation. Observations are normalized against records of *Betula pubescens* over the same period. Prevalence maps of *B. nana* in Finland are provided for comparison in Supplementary Fig. S1

**Fig. 2:**
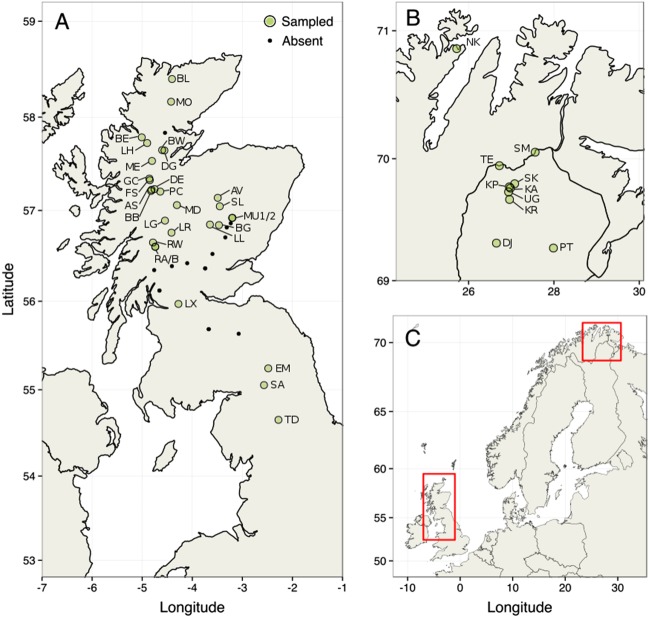
**a** Sampling locations in England and Scotland (Britain). **b** Sampling locations in Finland and Norway (Scandinavia). **c** Map of Northern Europe identifying study regions. Green circles are sampled populations. Black points denote populations from historical records that could not be relocated, and thus may be locally extinct

Based on distribution records (Fig. 1 and Supplementary Fig. S1), fresh tissue samples from 1220 *B. nana* individuals were collected from 29 populations across the species’ extant range in Britain and ten populations from northern Scandinavia, during the summers of 2012–2014 (Fig. 2). Plants were identified based on leaf morphology and growth structure, and sampling locations were chosen to encompass a similar geographic scale in both regions. All populations in Britain that we could locate were sampled, including exhaustive sampling of several small populations for which only one or two individuals remain (e.g., SA, TD). Attempts were made to minimize resampling of clonal or cryptic individuals by maintaining where possible a minimum distance of five meters between samples. Following guidelines agreed with partner organizations, care was also taken that the material removed amounted to <5% of the living plant to avoid lasting damage (See Supp. Mat. for more information on sampling). Due to difficulties in DNA extraction from *Betula* leaves, tissue samples consisted of small twigs 10–20 cm long. A section of cambium tissue from each sample was removed, dehydrated and stored on silica gel for DNA extraction. The remaining plant material from each individual was dried in a press and then stored on acid-free paper for future reference.

We collected field estimates of *Betula nana* census population size and assigned populations to the following categories: 1–10, 10–100, 100–1000 and 1000–10,000. Where possible, at populations with ~100 or fewer individuals, we counted all individuals.

### DNA extractions

Total genomic DNA was extracted from dried cambial tissue following modified cetyltrimethylammonium bromide (CTAB) protocol published in (Wang et al. 2013) and based on the approach of Doyle and Doyle (1987). The resulting DNA pellets were resuspended in 50–200 μl of TE buffer to normalize concentrations. Quality of samples was assessed using 1% gel electrophoresis and a Nanovue spectrophotometer (GE Healthcare, UK). Approximately 9% of samples were rejected due to degraded DNA, found mainly in the samples from Britain where small plant size limited the amount of tissue available. In all, 1115 DNA samples passed quality control from 39 populations. Nuclear microsatellite simple sequence repeats (PCR-SSRs) were amplified in all 1115 individuals and RAD sequencing was conducted in a subset of approximately 17% of individuals. Where samples were selected for RADseq, an additional 70% and 95% ethanol wash was added as a final stage before desiccation and rehydration in TE buffer.

### PCR-SSR development and genotyping

We used both previously published (Kulju et al. 2004; Truong et al. 2005; Gürcan et al. 2010) and de novo PCR-SSR primers (Supplementary Table S1). Novel primers were developed using the v4 Dwarf birch genomic resource (http://birchgenome.org/) (Wang et al. 2013). Di-nucleotide SSR motifs were identified using a local SequenceServer BLAST (Priyam et al. 2015). Scaffolds with high hit rates were identified and then transferred into the software WEBSAT (Martins et al. 2009) to enable the identification of repeats within the scaffold and the design of primer flanking sequences. Only one new PCR-SSR assay was developed per genomic scaffold in an effort to minimize linkage of markers. Six loci were developed for the plastid genomes.

A total of 24 PCR-SSR markers were selected and tested for stutter peaks and repeatability in a subset of 12 *B. nana* individuals from different populations in Scotland. Combinations of useable loci were then tested in silico using the software AutoDimer (Vallone and Butler 2004) to identify possible primer dimers or cross amplification. Multiplex panels (Supplementary Table S1) were run on a 2100 Bioanalyzer (Agilent Technologies, CA, USA) to confirm expected product sizes. Fragment length was determined by auto capillary gel electrophoresis on an ABI 3730xl (Applied Biosystems). Trace peaks were called manually using the software GeneMarker® (Softgenetics, LCC) using default quality control settings. The six plastid loci (Supplementary Table S1) were found to be largely monomorphic (>0.99) and were excluded from further analysis.

The PCR-SSR data were checked for presence of null alleles, deviation from Hardy-Weinberg equilibrium (HWE) and linkage-disequilibrium (LD) using the software GENEPOP v4 (Rousset 2008), with tests adjusted for multiple comparisons using the FDR method in the base function *p.adjust*. Loci were tested for signatures of selection using the method of Beaumont and Nichols (1996) implemented in the software Lositan (Antao et al. 2008) over 50,000 iterations. Putative clones were identified in the software Poppr (Kamvar et al. 2014).

### RAD sequencing, marker discovery, and genotyping

A subset of 190 individuals from 36 populations were selected for RAD sequencing. We used the cleavage enzyme *PstI*, as previous analysis of the draft *B. nana* genome identified an appropriate number (70,954) of cut sites (Wang et al. 2013). Cut DNA was normalized and submitted to FLORAGENEX (Portland, Oregon) for generation and sequencing of RAD tags following the protocol of Baird et al. (2008) and Hohenlohe et al. (2010). In brief, libraries were prepared with 96 unique 8-base barcodes, ligated via adapters to *PstI* digested genomic DNA. The resulting fragments were sequenced in two lanes of an Illumina GAIIx platform with single-end 1 × 100 bp chemistry. A single internal control sample generated from *Saccharomyces bayanus* was included in each lane.

Raw libraries were demultiplexed and barcode sequences checked for errors using the *process_radtags* module of Stacks v1.35 (Catchen et al. 2011, 2013). We used a quality threshold Phred score of ten in sliding windows of 15 bp. Three individuals failed and were removed due to low coverage. Reads were aligned to the *B. nana* genome and adapter sites trimmed using the software Bowtie2 (Langmead and Salzberg 2012). The mean number of retained reads per individual was 1,397,192 (Supplementary Table S2). SNP calling was performed using a bounded model in the *ref_map.pl* pipeline of Stacks v1.35, with two nucleotide mismatches permitted (-n 2), identified as an optimum threshold in Ilut et al. (2014) and a minimum stack depth of five (-m 5). Calls were corrected using the *rxstacks* module with a minimum log likelihood filter of -10 to retain a catalog locus (--lnl_lim -10) and other parameters set to default. Subsequently, *cstacks* and *sstacks* were manually rerun to rebuild and match to the catalog.

The *populations* module of the software Stacks (Catchen et al. 2013) was used to filter loci based on the following criteria. Loci were retained that were present in ≥70% of individuals in either of the sampling regions separately, or ≥70% of individuals across both regions combined. The final seven bases of each read were trimmed, as these showed a moderately higher frequency of SNPs possibly due to sequencing error. Putative SNPs from base 28 in all RAD loci were also removed, as the number present was highly elevated (>10×) indicating a possible error in one of the plate cycles.

For downstream demographic and Bayesian clustering analyses, polymorphisms with a minor allele frequency (MAF) of <0.01 were excluded. Similarly, to reduce LD these data were filtered to retain only a single locus per scaffold or contig in the *B. nana* genome assembly and achieve an r2 value of <0.2. We subsequently divided SNP loci into transition (RAD-SNP_ti_; *n* = 4775) and transversion (RAD-SNP_tv_; *n* = 3306) datasets based on the type of polymorphism recorded and analyzed these independently. Finally, SNPs were exported to other analysis software using custom scripts and PGDSpider 2.1.0.1 (Lischer and Excoffier 2012).

### RAD-SSR marker development

To generate RAD-SSR markers we filtered raw Illumina RADseq reads for SSR motifs by searching the consensus catalog for loci with di-nucleotide repeats (see Supplementary Information for reasoning). Initial filtering required loci to be present in >50 individuals. Reads were trimmed in the same manner as previously described and the frequency of each sequence string calculated (at least three identical reads were required for inclusion, and a minimum of ten reads per individual). Where a single sequence was present in an individual it was considered to be homozygous, where a second sequence was recorded in >10% of reads, it was called as a heterozygote (see Data Archiving for script details). Alleles were designated integer values unrelated to allele length or frequency. RAD-SSR markers were further screened for clones as described for PCR-SSR markers. For RAD-SSRs, unlike PCR-SSRs where alleles are distinguished on the basis of amplicon length, full sequence reads permitted us to distinguish alleles that differed in sequence but are the same length.

### Characterizing genetic diversity

Population allelic richness (*A*_r_) estimates were rarefied to allow correction for uneven sample size and estimated in ADZE (Szpiech et al. 2008) (Supplementary Fig. S2). Expected heterozygosity (*H*_e_) was calculated across all populations and markers by region in Hierfstat (Goudet 2005) and Adegenet 2.1.1 (Jombart 2008). Population-based *F*_IS_ was estimated across all markers using the method described in Catchen et al. (2013). For SSR markers, regional *F*_IS_ was also co-estimated with null allele frequency using INEst (Chybicki and Burczyk 2009). Global F_ST_ was estimated by marker and region in GENEPOP (Rousset 2008).

Some statistics characterize genetic patterns that may have accumulated over long time periods, such as the local genetic diversity (heterozygosity) or differentiation among populations (*F*_ST_). In order to investigate the relative importance of recent genetic drift in generating these patterns, we required an estimate of the current effective population size, (*N*_e_), at each location. An estimate was obtained using the LD-based method of Do et al. (2014) in NeEstimator V2. This approach was cross-validated by comparison with field estimates of *B. nana* census population size (see above).

Finally, we report an estimate of selfing rate (David et al. 2007) and assess population structure using bayesian clustering analysis, principal component analysis, and allele frequency spectrum plots (Supplementary Materials; Figs. S3–6, S8) for each marker and region.

### Population differentiation

Two different approaches were employed to assess population differentiation. In both cases we filtered for <25% missing data, genotypes in every population, and a minor allele frequency >0.05. First, pairwise *F*_ST_ between all population combinations was computed in Arlequin v3.5.2 (Excoffier and Lischer 2010) with significance tested over 10,000 iterations. Geographic distance matrices were calculated using Geographic Distance Matrix Generator V1.2.3 (Ersts, accessed 11-02-2016). Evidence for isolation by distance and regional discontinuities in genetic diversity were assessed using Mantel tests within fragmented and non-fragmented regions, performed in *vegan* (Dixon 2003). To linearize the relationship with difference, the statistic *M* was obtained as *M* = (1 – *F*_ST_)/*F*_ST_, and then plotted against log transformed geographic distance.

Second, to quantify the genetic divergence of each population from the regional mean, an alternative estimate of *F*_ST_ was defined; here termed as Maximum Likelihood *F*_ST_ (ML-*F*_ST_). Although *F*_ST_ is often thought of as a statistic describing the degree of genetic differentiation among a group of sub-populations, it can also be expressed in this way, since it can be formulated as a genetic correlation within a particular subpopulation (Balding 2003). We estimated the maximum likelihood value of local *F*_ST_ (for a given focal population) relative to the regional mean, using the multinomial Dirichlet likelihood function proposed by Balding and Nichols (1995; example script at https://github.com/qmwugbt112/FstCalc). Finally, to evaluate the influence of sample size, we estimated ML-*F*_ST_ across all loci from a single individual drawn from each population.

### Demographic history of dwarf birch

To infer the evolutionary and demographic history of dwarf birch in Britain since colonization, we used coalescent-based approximate Bayesian computation (ABC) implemented in DIYABC v2.0 (Cornuet et al. 2014). While there are a substantial number of possible scenarios and variables, we limited our analysis to three models addressing our core questions, as overly numerous or complex scenarios can result in poor parameter estimates (Bertorelle et al. 2010).

We performed our analysis in two steps. Initially, we used a pair of simple models, with a single contemporary British meta-population *N*_e_ variable (*NeBr*), to test for evidence of a historic bottleneck immediately on colonization (Scenario 2) or multiple bottlenecks after population differentiation (Scenario 1), potentially as a result of recent anthropogenic impacts. Subsequently, we incorporate census population information into the best-supported scenario (Scenario 1b), permitting independent British population *N*_e_ values to refine our posterior estimates. We test this against the null meta-population scenario from the previous analysis (Scenario 1). To further reduce the complexity of the analysis, we subsampled our full dataset (similar to the approach of Tsuda et al. 2015), retaining nine representative well-spaced populations from across the British distribution. For each marker set, this reduced dataset was tested together with an out-group formed from three Scandinavian populations from the center of our sampling distribution.

In all cases we generated 10^6^ simulations for each scenario across each marker set. Choice of summary statistics differed across marker sets largely due to their inherent characteristics; for example RAD-SSRs are incompatible with allele length or variance statistics due to the fact they are coded as unique integers. Summary statistics together with prior parameters are reported in Supplementary Tables S3–5. To evaluate our results, we checked that our observed data fell within the range of simulated datasets using principal component analysis. Once satisfied, the posterior probabilities of scenarios were estimated using logistic regression on the 1% of simulated datasets closest to the observed data. The robustness of model choice was further assessed by testing whether the observed data fell within 1000 simulated posterior datasets. Similarly, the bias and precision of parameter estimates were assessed with 1000 pseudo-observed test datasets drawn from posterior distributions, from which the difference between each point estimate and the true value was calculated. Finally, posterior parameter estimation was performed for the best-supported scenario on the 1% of closest datasets, and the information combined across marker sets into a single density distribution.

## Results

Historical records showed fragmentation and decline in the range of *B. nana* in the Britain over recent decades (Fig. 1). By contrast, in Scandinavia *B. nana* occurs abundantly in continuous populations (Supplementary Fig. S1). A total of 29 populations encompassing the extant British range and 10 Scandinavian populations were sampled (Fig. 2). In Britain it is notable that several populations that can be found in historical records, particularly those at the Southern range edge, could not be relocated during our fieldwork. Several that could be relocated had a total population size of five or fewer individuals. After quality control of DNA extractions, 1115 individuals were genotyped at 24 PCR-SSR loci, and a subset 190 individuals were retained for RADseq analyses (Table 1). Forty-nine individuals from the PCR-SSR dataset (4.4%) were subsequently identified as clonal genotypes and removed.

**Table 1:** *Betula nana* sampling locations, altitude, sample sizes, and census population sizes across the UK and Scandinavia

Population	ID	Latitude (°N)	Longitude (°E)	Altitude	PCR	RAD	Census size
*Scandinavia*							
Nordkapp	NK	70.863	25.722	10	30 (0)	6	10–100
Sirbma	SM	70.052	27.561	55	30 (4)	6	100–1000
Tenontie	TE	69.944	26.722	113	30 (0)	6	100–1000
Skalluvaara	SK	69.798	27.08	217	32 (0)	6	100–1000
Kevo Plateau	KP	69.773	26.955	318	32 (0)	6	100–1000
Kevojarvi	KA	69.763	26.981	124	31 (2)	6	100–1000
Gearddosjavri	UG	69.732	26.937	121	23 (2)	6	1000-–10,000
Kevo Reserve	KR	69.671	26.96	218	31 (0)	6	1000–10,000
Kotilampi	DJ	69.313	26.653	216	29 (0)	6	100–1000
Partakko	PT	69.272	27.988	124	28 (0)	6	1000–10,000
				Sub-total	296 (8)	60	
*Britain*							
Ben Loyal	BL	58.401	–4.404	300	30 (0)	6	10–100
Meall Odhar	MO	58.163	–4.423	404	25 (11)	6	25
Beinn Enaiglair	BE	57.786	–5.009	480	27 (0)	6	10–100
Luichart	LH	57.725	–4.9	268	32 (2)	6	10–100
Ben Wyvis W	BW	57.65	–4.602	482	34 (0)	6	100–1000
Ben Wyvis E	DG	57.646	–4.556	472	21 (0)	-	10–100
Loch Meig	ME	57.534	–4.804	450	26 (0)	6	10–100
Glen Cannich	GC	57.345	–4.856	455	33 (0)	6	66
Faskanyle	FS	57.327	–4.848	486	31 (0)	-	100–1000
Dundreggan	DE	57.231	–4.754	448	30 (0)	6	38
An Suidhe	AS	57.224	–4.812	661	30 (2)	3	10–100
Beinn Bhreac	BB	57.211	–4.823	500	33 (0)	6	50
Portclair	PC	57.204	–4.639	478	41 (5)	6	41
River Avon	AV	57.137	–3.491	549	31 (0)	6	60
Monadhliaths	MD	57.058	–4.307	712	33 (5)	6	10–100
Meall an t’slugain	SL	57.045	–3.451	633	31 (0)	6	10–100
Loch Muick E	MU1	56.92	–3.198	492	31 (0)	6	1000–10,000
Loch Muick W	MU2	56.918	–3.205	517	32 (0)	6	1000–10,000
Loch Laggan	LG	56.889	–4.545	364	49 (6)	6	49
Loch Loch	LL	56.846	–3.647	673	30 (4)	6	10–100
Ben Gullabin	BG	56.84	–3.467	594	5 (0)	1	5
Loch Rannoch	LR	56.758	–4.415	499	29 (2)	6	29
Rannoch West	RW	56.65	–4.785	306	31 (2)	6	1000–10,000
Rannoch Moor B	RB	56.603	–4.74	304	31 (2)	6	100–1000
Rannoch Moor A	RA	56.603	–4.738	295	30 (0)	-	100–1000
Lennox	LX	55.97	–4.276	164	9 (0)	2	9
Emblehope	EM	55.244	–2.483	448	2 (0)	1	2
Spadeadam	SA	55.053	–2.568	275	1 (0)	1	1
Teesdale	TD	54.654	–2.28	499	2 (0)	2	2
				Sub-total	770 (41)	130	
				Total	1066 (49)	190	

PCR gives sample sizes for PCR-SSR data. RAD gives sample sizes for RAD-SSR, RAD-SNP_ti_, and RAD-SNP_tv_ datasets. Values in parenthesis are the number of putative clones identified

### PCR-SSR marker validation

The number of PCR-SSR alleles per nuclear locus ranged from two to 42, with 446 alleles in total (Supplementary Table S1). Four loci were found to deviate consistently from HWE across populations, most likely due to null alleles. Of these, two were also identified as putatively under positive selection, thus all were excluded from analysis of genetic differentiation.

### RAD-SNP discovery

A total of 339,291,129 reads were generated from two single-end Illumina HiSeq lanes, of which 81.66% passed strict quality controls. Three individuals were removed due to low sequencing coverage. The number of reads retained per individual ranged from 373,727 to 2,852,627, with a mean of 1,397,192 (Table S2). Across all individuals an average of 78.3% of reads aligned to the *B. nana* reference genome. Percentage alignment was consistently higher in Scandinavian samples, despite the reference genome being of Scottish provenance. We attribute this to poorer quality DNA derived from extractions on Scottish samples, due to the smaller size of twigs and difficulties in removing all bark from cambium tissue. Despite approximately 20% of reads that did not align being excluded, the reference-mapped pipeline identified approximately a third more usable loci than when the analysis was undertaken de novo (data not shown). This is perhaps due to reference-based mapping performing better at distinguishing paralogs. A threshold of 70% presence resulted in 6092 loci containing 23,882 SNPs. Quality filtering retained 17,694 SNPs with an overall Transition/Transversion ratio of (11:10). With minor alleles occurring at a frequency of <0.01 removed, 4208 loci and 8081 SNPs were retained, of which 4775 were transition and 3306 transversion polymorphisms (144:100); thus proportionately more of the low frequency polymorphisms were transitions.

### RAD-SSR discovery

Simple sequence repeat motifs were initially identified in 3507 RADseq loci, with 196 loci meeting initial filtering criteria. We excluded three further loci with >40 unique genotypes due to the risk of a high number of sequencing errors in these reads. The number of alleles per locus ranged from 2 to 31 with a total of 1758 alleles across all populations. No evidence of clones was found in any of the RADseq samples.

### Marker comparison

The number of alleles across all samples ranged from 414 in PCR-SSRs to 9428 in RAD-SNP_ti_. Data are summarized by marker and region in Table 2. Despite fewer loci, PCR-SSRs reported a greater number of alleles per locus than RAD-SSRs. Across all datasets, patterns of allelic richness (*A*_r_) and expected heterozygosity (*H*_e_) displayed a similar pattern (Table 2), being highest in the fastest mutating markers. Inbreeding estimates (*F*_IS_) were lowest in SNPs datasets, and higher among SSR data, with RAD-SSR data reporting the highest values. As expected, SSR marker *F*_IS_ values were lower when co-estimated with null allele frequency.

**Table 2:** Summary statistics for the four different marker sets used in this study, reported by region, including: sample size; number of loci: number of alleles (nAll); allelic richness (*A*_r_) rarefied to 20 individuals; expected heterozygosity (*H*_e_); fixation index (*F*_IS_) with INEst estimation, which accounts for null alleles, in parentheses; and global *F*_ST_ by region (*F*_ST_) with 95% confidence interval

Marker	Ind.	Pop.	Loci	nAll	*A*_R_	*H*_e_	*F*_IS_ (INEst)	*F*_ST_ (95% CI)
*Scandinavia*								
PCR-SSR	296	10	18	323	5.695	0.712	0.206 (0.057)	0.021 (0.010–0.068)
RAD-SSR	60	10	193	1177	3.961	0.425	0.273 (0.147)	0.021 (0.000–0.028)
RAD-SNP_ti_	60	10	4714	8383	1.437	0.118	0.148	0.019 (0.013–0.022)
RAD-SNP_tv_	60	10	3299	5836	1.425	0.117	0.146	0.018 (0.013–0.024)
*Britain*								
PCR-SSR	751	24	18	369	5.479	0.677	0.179 (0.065)	0.076 (0.066–0.094)
RAD-SSR	120	21	193	1224	2.674	0.342	0.280 (0.209)	0.054 (0.044–0.081)
RAD-SNP_ti_	120	21	4714	8839	1.434	0.111	0.122	0.089 (0.084–0.094)
RAD-SNP_tv_	120	21	3299	6144	1.426	0.108	0.119	0.092 (0.086–0.098)

Data by population is reported in Supplementary Tables S6 and S8. In the UK, populations with less than five individuals were excluded from these statistics

### Regional patterns of genetic diversity

Summary statistics comparing genetic diversity among the British and Scandinavian populations are shown in Table 2 (see also Supplementary Figs. S3 and S7). The five smallest British populations are excluded from these measures as they only had one or two individuals. Allelic richness and expected heterozygosity was significantly higher in Scandinavia than in Britain for all markers (Supplementary Table S6; Two sample *t*-test, all *p* ≤ 0.01), with the exception of PCR-SSR *H*_e_, which displayed the opposite trend. Fixation indices were higher in Scandinavia than in Britain for both RAD-SNP datasets (Two sample *t*-test, all *p* < 0.01). For SSR markers, fixation indices were not significantly different in Scandinavia and Britain, and null alleles affected fixation index estimates more in Scandinavia than in Britain, likely because our markers were first developed using British populations. Global *F*_ST_ was significantly higher in Britain across all RAD markers, with 95% confidence intervals only weakly overlapping in PCR-SSR markers. The rather small difference in overall levels of genetic diversity in Scandinavia and Britain is reflected in the diversity data for individual populations (Supplementary Table S6 and Supplementary Fig. S7). Larger British populations had similar levels of genetic diversity within them as Scandinavian populations.

**Fig. 3:**
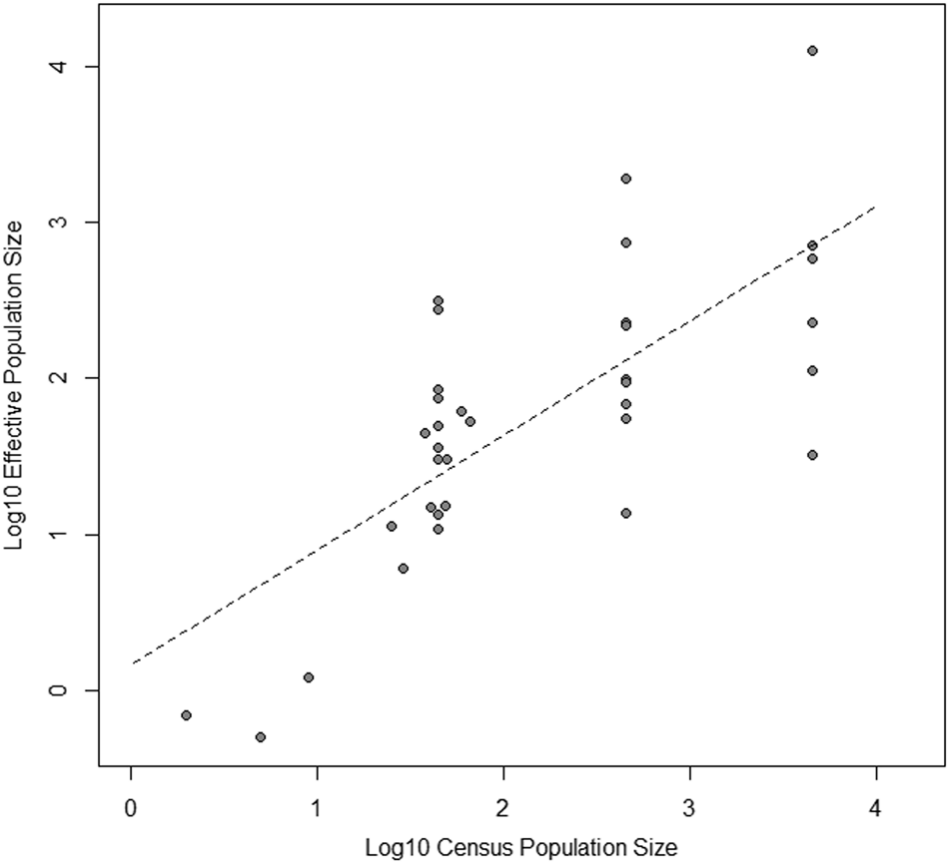
Plot of census population size vs. LD-based effective population size estimates for all populations

Among both British and Scandinavian populations, LD-based estimates of effective population size were similar to census population sizes and showed a highly significant relationship (*F*_1,33_ = 37.12, *p* < 0.0001; Fig. 3; data are presented in Supplementary Table S8). Interestingly, we also find evidence for selfing in British populations (*s* = 0.04 [0–0.26]), but much less so in Scandinavia (*s* = 0.02 [0–0.17]) (see Supplementary Materials; Supplementary Table S7).

### Population differentiation

**Fig. 4:**
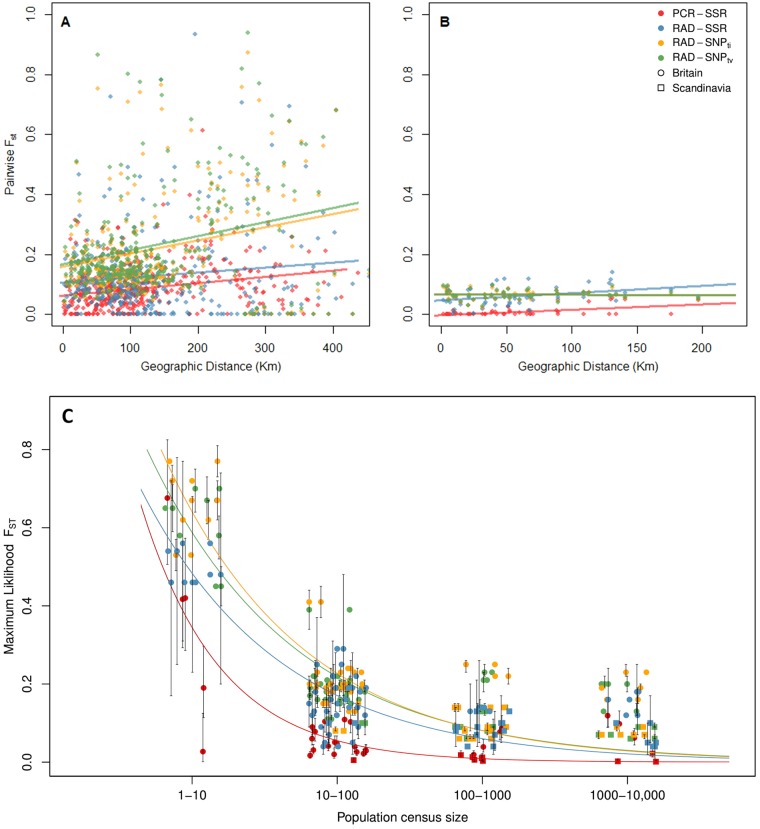
**a**, **b** Scatter plot of linearized pairwise *F*_ST_ vs. pairwise geographic distance for all study populations for Britain (**a**) and Scandinavia (**b**). Weak isolation by distance (*p* < 0.05) was detected in Britain and Scandinavian populations using PCR-SSRs. All other relationships were non-significant. **c** Plot of census population size estimates and maximum likelihood *F*_ST_, for each marker type, using all datasets across both regions. Noise has been added to each of the four *x*-axis categories to facilitate visualization

Overall we found little evidence for isolation by distance (IBD) across markers, RAD-SSRs (Britain: *r* = –0.003, *p* = 0.46; Scandinavia: *r* = 0.36, *p* = 0.055), RAD-SNP_ti_ (Britain: *r* = –0.003, *p* = 0.06; Scandinavia: *r* = –0.03, *p* = 0.51) and RAD-SNP_tv_ (Britain: *r* = 0.16, *p* = 0.10; Scandinavia: *r* = 0.002, *p* = 0.45), except in PCR-SSRs (Britain: *r* = 0.28, *p* = 0.009; Scandinavia: *r* = 0.34, *p* = 0.044) (Fig. 4). Furthermore, in all cases small *r* statistics suggest that only a very limited proportion of genetic divergence is explained by distance. To confirm that differing PCR-SSR results are not due to larger sample size, we also report mantel test results with populations subsampled to six individuals (Britain: *r* = 0.22, *p* = 0.043; Scandinavia: *r* = 0.05, *p* = 0.42).

The population estimates of ML-*F*_ST_ were significantly higher in Britain compared to Scandinavia across all markers (Two sample *t*-test, all *p* < 0.01). This is further supported by STRUCTURE analysis, which shows a stronger pattern of population structure in Britain compared to Scandinavia (Supplementary Figs. S4–5). ML-*F*_ST_ values were negatively correlated with latitude in Britain (PCR-SSR: *F*_1,27_ = 13.91, *p* < 0.001; RAD-SSR: *F*_1,24_ = 19.37, *p* < 0.001; RAD-SNP_ti_: *F*_1,24_ = 24.92, *p* < 0.001; RAD-SNP_tv_: *F*_1,24_ = 27.96, *p* < 0.001), but showed no significant correlation in Scandinavia (Supplementary Table S8). When ML- *F*_ST_ was plotted against both census and LD-based estimates of effective population size there was a strong negative relationship for all markers (Fig. 4 and Supplementary Fig. S9). We further discriminated against sample size artifacts by performing the analyses with a single individual drawn from each population, which also displayed a negative relationship (Supplementary Fig. S10). The data clearly show a strong trend for markers with higher mutations rates to exhibit lower ML-*F*_ST_ values.

### Demographic history

**Fig. 5:**
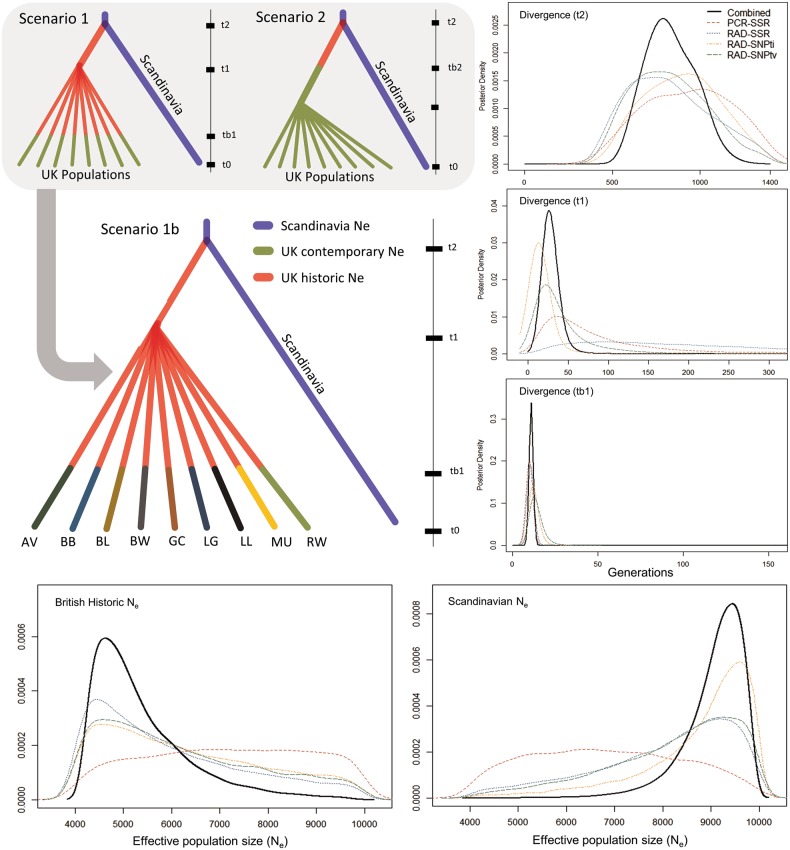
Dendrograms showing proposed scenarios to describe the evolutionary history of dwarf birch after the last glacial maximum. Line charts show posterior demographic density distributions for scenario 1b across all marker sets independently (dashed lines) and with information combined (black lines)

Coalescent modeling in DIYABC showed all marker sets support Scenario 1 over Scenario 2 (Fig. 5 and Supplementary Fig. S11), suggesting that the losses in genetic diversity in British populations mainly occurred in recent bottlenecks in differentiated local populations, rather than upon post-glacial colonization of Britain. We then tested a scenario (1b) which allows population sizes to change at some time point post-fragmentation. In this model, we included a diffuse prior on each population’s *N*_e_ centered on our census size estimates. Both RAD-SNP marker sets supported scenario 1b over scenario 1 (Supplementary Fig. S11), but the RAD-SSRs and PCR-SSRs did not strongly support one of these models over the other.

We estimated combined posterior parameter distributions based on scenario 1b. These supported differentiation of British and Scandinavian lineages t2 = 789 (CI = 521–1274) generations ago, presumably shortly after colonization of Britain; an event that would have substantially limited gene flow. The estimates suggest that scandinavia maintained a somewhat larger historic Ne (NeSc = 9384, CI = 4800–9826) than Britain (NeBr = 4650, CI = 4194–9494), although their confidence intervals overlap substantially. Subsequently, British populations appear to have become differentiated very recently at t1 = 23 generations ago (CI 10–415). The *N*_e_ estimates for local British populations fitted well with our census estimates and LD *N*_e_ estimates (Table 3 and Fig. 3), a result which is not due to limitations set by the priors, with the exception that the MU estimate was close to its minimum prior value (50) for all markers (the LD *N*_e_ estimate was 715 and census estimate was up to 2000). Model checking, bias and precision estimates in these data are reported in Supplementary Table S9 and Supplementary Fig. S12.

**Table 3:** Posterior demographic parameter estimates with 0.05–0.95 confidence intervals for each marker based on approximate Bayesian computation analysis

Parameter	SSR		RAD-SSR		RAD-SNP_ti_		RAD-SNP_tv_		Combined	
	Peak	CI (0.05–0.95)	Peak	CI (0.05–0.95)	Peak	CI (0.05–0.95)	Peak	CI (0.05–0.95)	Peak	CI (0.05–0.95)
tb1	10	(10–10)	11	(10–12)	12	(10–17)	13	(10–21)	11	(10–17)
t1	45	(17–308)	99	(39–632)	14	(10–32)	27	(12–132)	23	(10–415)
t2	1021	(545–1314)	757	(488– 1260)	932	(559–1254)	765	(515–1203)	789	(521–1274)
NeBr	6884	(4429–9703)	4547	(4133– 9110)	4668	(4183–9332)	4701	(4178–9320)	4650	(4194–9494)
NeSc	6399	(4412–9396)	9143	(5148– 9774)	9475	(6209–9909)	9234	(5457–9845)	9384	(4800–9826)

Final column indicates estimates using combined information from all marker distributions

*tb1* time of bottleneck in British populations, *t1* time of divergence of British populations, *t2* time of divergence of British and Scandinavian populations, *NeBr* British historic (pre-bottleneck) effective population size, *NeSc* Scandinavian effective population size

## Discussion

We generated population genomic datasets for dwarf birch populations in Britain and Scandinavia using four types of markers: 18 PCR-SSR loci with a total of 414 alleles, 193 RAD-SSR loci with 1758 alleles, 4775 RAD-SNP_ti_ biallelic loci and 3306 RAD-SNP_tv_ biallelic loci. We successfully genotyped 1066 individuals with the PCR-SSRs and 187 individuals with the RAD-based markers. The number of loci, alleles and samples for these marker sets reflect their abundance in the genome, the dynamics of their mutation and the ease of assaying them.

Overall, the four different marker types showed similar patterns among populations and regions, despite considerable differences in marker numbers and sample sizes. Estimates of most diversity measures showed a clear pattern of PCR-SSR > RAD-SSR > RAD-SNP_ti_ > RAD-SNP_tv_. Figures 4 and 5 (and Supplementary Figs. S7 and S9) show that population differentiation measured by *F*_ST_ is higher for the SNP markers than in the PCR-SSR markers, with the RAD-SSRs being intermediate. We expected all markers to give the same *F*_IS_ estimates, as nonrandom mating should affect all markers equally. However SSR based markers reported slightly higher *F*_IS_ values, which can be explained by our higher estimates of null allele frequencies, particularly in Scandinavia (Table 2 and Supplementary Fig. S6).

### Conservation genetic status of dwarf birch populations in Scotland

Overall, we found lower allelic richness and expected heterozygosity in Britain than in Scandinavia, and higher population differentiation. We also find a weak signal of an increased selfing rate in the UK (Supplementary Table S7). Genetic diversity was lowest in the British southern trailing range edge where effective population sizes were low (Table 2 and Fig. 3), whereas larger British populations were intermediate. In the medium-term population subdivision and differentiation may maintain genetic diversity in Britain, despite reduced *N*_e_, until local populations become too small.

In Scandinavian populations, *F*_ST_ estimates are smaller and there is little evidence of isolation by distance—hence gene flow appears to be moderating the accumulation of genetic differentiation (Meirmans 2015). In Britain, *F*_ST_ estimates are greater and vary substantially, so it might seem surprising that there is again little evidence of isolation by distance. Instead the higher *F*_ST_ values particularly for Southern populations suggest that these range edge populations have become isolated and increasingly differentiated over time, while central populations show patterns of lower differentiation, which is more consistent with Scandinavia.

The greater differentiation of some populations shows that dwarf birch can be vulnerable to population fragmentation, even though it shares some attributes with larger trees that could provide resilience (Lowe et al. 2015), such as longevity and wind-dispersed pollen. These traits could help maintain genetic diversity over longer times and share it over long distances. However, our plots of *F*_ST_ against the current census population size (Fig. 5; also see Supplementary Fig. S9 for a plot with linkage-disequilibrium based estimates of *N*_e_), show that differentiation is clearly related to current population sizes with a strong effect in populations currently around ten individuals. This effect is also seen in loss of allelic richness values in smaller populations (Supplementary Table S6). These results suggest that the populations have been at the current population sizes for sufficient time to affect the genetic diversity—of the order of the same number of generations as the population size. This relationship between the effective population size and census size was also seen in the ABC analysis, and is relative unusual in population genetic surveys (Frankham 1995; Vucetich et al. 1997). Part of the explanation could be in the relatively small population sizes for many populations, meaning that recent population history dominates the genetic patterns.

### The history of British population structure

The combined information from the four marker datasets supported a scenario, in the ABC modeling in which the distribution of genetic diversity could be explained by the recent population size of fragmented populations, with the subdivision into smaller populations dating a long time after colonization of Britain. The RAD-SNP datasets provided more information about differences in individual population *N*_e_, favouring different population sizes when analyzed separately.

The ABC models give estimates of the timing of events in number of generations rather than years, so their interpretation requires an estimate of generation time. *Betula nana* as individuals can set seed in as little as 18 months in a glasshouse (personal observation), setting a lower bound on generation time. A more central estimate can be obtained if we assume divergence occurred between the ancestral British and Scandinavian dwarf birch populations (t2 = ~789 generations) just after the last ice age 10–12,000 years ago. Dividing by the number of generations gives a generation time is 10–15 years, which is biologically plausible. This calibration would place the divergence among British populations at 230–345 years ago (t1 = ~23). That would date the ABC estimate for the time of current population subdivision to a the period when large scale anthropogenic activity including lowland agriculture had begun to shape the distribution of woody plants in Scotland (Smout et al. 2005; Godwin 1975; Tipping 1994).

The estimates for the date of reduction in current population size must be treated with more caution, since for time periods that are short compared to the mutation rate similar genetic patterns can be produced by longer times at larger population size, or shorter times at smaller. The partial confounding of these parameters explains why we did not have power to estimate different times for each fragment (data not shown). Taken at face value, the estimate of 11 generations (tb1 = ~11) is sufficient for the very smallest populations to have lost a substantial part of their genetic variation, and corresponds to 110–165 years. Accelerated forest clearance is known to have occurred in the sixteenth to nineteenth century, combined with a shift to livestock farming during the highland clearances (Dodgshon and Olsson 2006) and an increase in wild herbivores, partly as a result of the removal of wild predators (Holl and Smith 2007); these could plausibly have impacted dwarf birch numbers. This is also consistent with survey data showing substantial recent declines, presented in Fig. 1. One of populations, MU, produced an estimate of recent population size at the lower limit of its prior distribution. This disparity may be explained by its location: lowland and Southern populations are likely to have gone through earlier reductions in population size—which would be recovered in our model as a smaller *N*_e_ estimate.

### Implications for dwarf birch conservation

Our results suggest that the long-term prospects for many dwarf birch populations in Scotland may be good, as there are levels of genetic diversity comparable to Scandinavia, despite fragmentation and reduced population size. Our ABC models suggest only moderate loss of diversity in the British populations upon post-glacial colonization, and that losses of diversity since then occurred in localized populations during their divergence from one another. Thus, different sub-sets of the ancestral variation of British *B. nana* populations are preserved in different fragments, so that overall levels of diversity in British *B. nana* populations are only moderately lower than the large continuous populations of Scandinavia. The long-term viability of *B. nana* in Britain may be enhanced by artificially restoring gene flow between populations, perhaps by culturing seedlings within, or transplanting among populations (Aitken and Whitlock 2013). Even very modest interventions may be effective.

The very smallest populations have diverged in allele frequency and lost genetic diversity at the genome-wide marker loci we surveyed. We can explain these effects as a consequence of recent severe isolation and reductions in effective population size. We would, therefore, expect loss of alleles conferring adaptive variation and inefficient selection to maintain traits at the local adaptive optimum. Consequently these populations are unlikely to harbor adaptive rear-edge diversity that needs to be conserved (*sensu lato* Hampe and Petit 2005). We, therefore, suggest that these populations could be enhanced by transplantation from the larger populations, but that it is unlikely to be beneficial to the larger populations to receive assisted gene flow from these small populations.

### Broader utility of our approach

While, the potential for orders of magnitude more data from SNP analyses based on modern NGS technology is widely appreciated, comparative studies combining different categories of population genetic markers remain sparse (examples include Bradbury et al. 2015; Jeffries et al. 2016; Hodel et al. 2017). Here, we have found that for studies focusing on putatively neutral differentiation and drift, a modest number of SSR markers produce results that are comparable with thousands of SNPs (see also Hodel et al. 2016). We suggest that RAD-SSRs could be more widely used as they overcome many of the limitations of PCR-SSRs, yielding a high mutation-rate marker set, with >4 possible alleles per locus, and a well-specified ascertainment process that does not entail additional sequencing. In this way, we were able to exploit markers with different mutation rates to explore different demographic scenarios. Furthermore, comparing different types of markers can alert the researcher to biases introduced by the different ascertain processes (in our case possible effects on demographic estimates of contemporary population size and inbreeding), and evolutionary processes occurring at different rates. For conservationists where management interventions based on population data may influence the fate of species, robust conclusions based on multiple independent datasets are advisable.

### Data archiving

- Raw PCR-SSR data available from the Dryad Digital Repository: 10.5061/dryad.v75rj24.
- Illumina read data from RADseq libraries has been uploaded to the European Nucleotide Archive project PRJEB26807, sample accessions ERS2598190- ERS2598376
- Newly developed PCR-SSR primers are reported in the Supplementary Materials
- RAD-SSR marker data mining script available on Github: https://github.com/JamesBorrell/RAD-SSR_extraction_scripts.git
- Maximum Likelihood *F*_ST_ function available on Github: https://github.com/qmwugbt112/FstCalc

## Electronic supplementary material

Supplemental Material
