# Supplementary material for: Genetic diversity maintained among fragmented populations of a tree undergoing range contraction
Source: Heredity (Edinb). 2018 Aug 15;121(4):304–18. doi: 10.1038/s41437-018-0132-8 (PMC6134035; doi:10.1038/s41437-018-0132-8)
Supplement: Supplementary file 1 — Supplemental Material [file 41437_2018_132_MOESM1_ESM.docx]

**Supplementary Material**

Field sampling and tissue collection 1

Census population estimates 2

Selfing analysis 2

NGS-SSR di-nucleotide repeats 2

Rarefied allelic richness estimates 3

Maximum likelihood F_ST_ 3

Analysis of population structure 4

Principal Component Analysis 6

ABC scenario choice and summary statistics 6

Supplementary Tables 8

Supplementary Figures 18

Supplementary References 30

Appendix 1: Maximum Likelihood F_ST_ Script 31

## Field sampling and tissue collection

A minimum of five meters between samples was used to avoid accidental resampling of the same clonal individual. This was based on Autumn observations where slight changes in phenology of leaf senescence permits identification of different individuals. Nevertheless, our method included genetic identification of clones at a subsequent step to ensure these were not included in population genetic analysis.

A maximum of 5% of the above ground living plant was collected to avoid damage. This protocol was based on advice from conservation partners in Scotland and is considered conservative based on grazing pressure across much of Scotland. Indeed, we also highlight that previous studies found 80% of plant biomass to be below ground in Betula nana (Chaplin, 1980).

## Census population estimates

To validate the relationship between genetic estimates of N_e_ and F_ST_, we conducted field estimates of *Betula nana* census population size. We assigned populations to the following categories: 1-10, 10-100, 100-1,000 and 1,000-10,000. Where possible, at populations with ~100 or less, we counted all individuals. For larger populations we used expert opinion to estimate greater than or less than 1000 individuals.

## Selfing analysis

To further investigate the evolution of fragmented dwarf birch populations and the high levels of F_IS_ reported in this study, we estimated population and regional selfing rates using the package RMES (David *et al.*, 2007). We used two methods; first we used both the g_2_ estimator and the Maximum Likelihood approach (LnL=0.0005, kmax=10) to estimate population selfing rates. Secondly we constrained the ML search to obtain a single overall selfing rate estimate for each region (Table S7).

## NGS-SSR di-nucleotide repeats

In this study we elected to only evaluate di-nucleotide repeats, as this minimized the overall base length of the microsatellite markers. We highlight that this approach may be easily applied to other, more complex repeat numbers and arrangements, but that this would however require longer NGS (e.g. RADseq) read lengths for the entire repeat motif to be unambiguously included within the overall sequence.

## Rarefied allelic richness estimates

To achieve broad population sampling whilst ensuring sufficient sample sizes, we used a randomization approach to simulate different sample sizes on rarefied allelic richness estimates. We calculated rarefied A_R_ as described in the main text for 1-12 individuals from Loch Muick where two locations were sampled within the same meta-populations. This was conducted over 10 iterations, with individuals randomly sampled without replacement (Figure S2). Results suggest that six individuals are sufficient to yield results similar to larger sample sizes.

## Analysis of population structure

To assess evidence of genetic structure in *B. nana* populations, Bayesian clustering was performed on each dataset in the software STRUCTURE (Pritchard, Stephens and Donnelly, 2000). STRUCTURE analysis was performed with the number of pre-assigned genetic clusters (K) ranging from one to eight, the LOCPRIOR model (Hubisz *et al.*, 2009), no-admixture and correlated allele frequencies assumed due to anticipated recent joint ancestry. Following the advice of Pritchard in Benestan et al. (2016) we opted to divide computing time over many independent shorter runs, rather than few long runs. This is because STRUCTURE converges fairly quickly but is poor at exploring alternative peaks in parameter space. Thus 50 independent runs were performed for each K, with 20,000 Markov chain Monte Carlo repetitions after 20,000 burn-in iterations and the behavior of the MCMC checked using the *acf* function in R. The posterior probability of K was estimated using the statistic ∆K (Evanno, Regnaut and Goudet, 2005), though we note its limitations (Falush, Dorp and Lawson, 2016), implemented in Structure Harvester (Earl and vonHoldt, 2012) and visualized using CLUMPAK (Kopelman *et al.*, 2015) and R software.

Inspection of Ln P(D) plots strongly indicated that the best-supported value of K was K = 2 for all markers sets (Figure S4). In the RAD-SNP datasets, the UK and Scandinavian regions were highly differentiated with confident correct assignment of all but one individual from Teesdale (TD) in the RAD-SNP_tv_ dataset. The PCR-SSR dataset largely discriminated between the UK and Scandinavia, except for a scattering of individuals, perhaps due to homoplasy. RAD-SSRs displayed much poorer cluster assignment with very few individuals being assigned to clusters with 100% support.

We expected all markers to give a similar discrimination between clusters in Bayesian structure analysis, separating UK and Scandinavian populations, with the possible exception of PCR-SSRs, given that these are more prone to homoplasy. However, we found RAD-SSRs to perform very poorly in this discrimination. The greater success of PCR-SSRs may have been due to a larger sample size than for the RAD-SSRs. Alternatively, the RAD-SSRs may have performed poorly due to higher levels of missing data, resulting from strict filtering to identify SSR loci. Another possibility is that real homoplasy is more common in our RAD-SSRs, given that they are based on shorter repeat lengths in which reverse mutations might be more common.

Subsequently, we performed STRUCTURE analyses separately on British and Scandinavian geographic regions for PCR-SSR and RAD-SNP_tv_ datasets. In each case, analysis was performed with the number of pre-assigned genetic clusters ranging from one to N+2 populations and the same parameters as previously described.

Substructure analysis within the UK identified 22 clusters (Figure S5B). Populations that were sampled in close proximity (e.g. populations MU1 and MU2, RA and RB) clustered together. Populations from around the Dundreggan estate, west of Loch Ness (e.g. BB, DE), tended to display less certain population assignment, possibly attributed to admixture or reduced differentiation in this relatively stable portion of the species’ UK range. Comparatively, RAD-SNP_tv_ based data indicated 12 clusters (Figure 5C), but provided less clear population assignment. Assessment of structure in Scandinavia weakly supported two clusters, but we note that the DeltaK method is not able to imply a result of k=1, which based on the high variability of the runs we believe to be the most biologically meaningful interpretation.

## Principal Component Analysis

To visualize the major axes of genetic variation, principal component analysis was performed on all four genetic data sets. Analysis was implemented using the package ‘adegenet’ (Jombart, 2008) and the R function ‘dudi.pca’ (R Development Core Team, 2014).

## ABC scenario choice and summary statistics

When modeling large numbers of wild populations over a long time period there may be a prohibitively high number of possible demographic scenarios meaning that a degree of initial selection must be undertaken. Furthermore there are risks or multiple testing or overfitting when performing a large number of analyses or testing highly complex models.

In this study we specifically sought to test the question “Can a combination of marker types distinguish whether the current distribution of genetic diversity in Britain is a result of recent fragmentation events or early patterns of colonization after the last glacial maximum?”. Thus to thin the initial number of plausible scenarios, and due to limitations on computing power, we performed shorter runs (10^5^ simulations) and inspected principal component analysis plots to assess whether observed data fell within the range of simulated data.

For each marker set we elected to use all appropriate summary statistics for estimation. However, due to different marker characteristics, these differed across the four analyses. In particular, due to the fact that RAD-SSRs were coded as unique integers it was not possible to use allele length or variance statistics such as ‘Mean size variance’ or ‘(du)_2_ distance’. Similarly, ‘number of allele’ based statistics for SNP markers are inappropriate as only bi-allelic markers are retained (see DIY-ABC manual for full explanation of summary statistics and references).

## Supplementary Tables

**Table S1.** Primer sequences, product lengths and general characteristics of 24 microsatellite SSR loci tested in this study.

| Origin | Genbank No. | Genome | Repeat | Forward Sequence | Reverse Sequence | Annealing Temp. | Predicted length | Product length | No. Alleles | Multiplex panel |
| --- | --- | --- | --- | --- | --- | --- | --- | --- | --- | --- |
| Kulju *et al.* 2004 | AF310875 | Nuclear | (GA)_6_TA(GA)_6_ | TGGTTGACGTGACGTTGATT | GGCCCATAGGGAAGATAAGC | 64 | 210–222 | 207-251 | 39 | Panel 1 |
| Kulju *et al.* 2004 | AF310856 | Nuclear | (AG)_4_AA | TTTCCAACGCTTTCTTGATG | TGGATAAGGAAGGGCATGTC | 64 | 152–206 | 166-201 | 30 | Panel 1 |
| Kulju *et al.* 2004 | AF310871 | Nuclear | (CA)_3_(GA)_14_ | CACCACCACAACCACCATTA | AACACCCTTTGCAACAATGA | 64 | 93–108 | 173-215 | 32 | Panel 1 |
| Kulju *et al.* 2004 | AF310851 | Nuclear | (CT)_13_A(TC)_6_ | CACACTGCTGCCTGA | TCATAAAACCCTCAAAGAAT | 64 | 134–166 | 209-217 | 8 | Panel 1 |
| Kulju *et al.* 2004 | AF310854 | Nuclear | (CT)_12_CCTT(CT)_4_ | GTTTTGGGTTTCCACTTCCA | ACTGGTAATACCTTTACCAAGCC | 64 | 146–152 | 139-154 | 14 | Panel 1 |
| Kulju *et al.* 2004 | AF310864 | Nuclear | (GT)_18_(GA)_14_ | GGGGATCCAGTAAGCGGTAT | CACACGAGAGATAGAGTAACGGAA | 64 | 178–226 | 195-211 | 16 | Panel 1 |
| Kulju *et al.* 2004 | AF310877 | Nuclear | (CT)_14_ | TCTACGCTGTGACCAGTC | AGAATCCTAGCCTTTTCAAT | 55 | 168–236 | 172-218 | 27 | Panel 2 |
| Truong *et al.* 2005 | AF310847 | Nuclear | (AG)_16_ | CGGGAAGATATGCAGTGTTT | TTGGCGGGTGAAGTAGAC | 58 | 208–252 | 205-217 | 8 | Panel 2 |
| Truong *et al.* 2005 | AF310848 | Nuclear | (CT)_9_ | CTATATTGGCTCCAAGCAC | ACACCCACACTGACAGATAA | 55 | 94–128 | 94-121 | 17 | Panel 2 |
| Truong *et al.* 2005 | AY423611 | Nuclear | (TC)_14_ | TGGCAGCACGAAAGT | TGGGAATGAGAGAACAAG | 48 | 172–210 | 176-218 | 35 | Panel 2 |
| Kulju *et al.* 2004 | AF310866 | Nuclear | (CT)_11_ | GGCCAACAGATATAAAACGACG | TTTTAAATGCCCACCTTCCC | 48 | 295–307 | 284-312 | 27 | Panel 2 |
| Mehlenbacher *et al.* 2010 | AY423613 | Nuclear | (AG)_17_ | CTTACCGTCCTGCCAAGGT | ACCACCACAGCCACAACC | 60 | 235 | 208-242 | 30 | Panel 2 |
| *De novo* | - | Nuclear | (CT)_5_TGCTGGC(T)_3_(CA)_7_ | TATCAGAGACCAATGCCCAAG | CCAGGGAGTGTAGACAAAGGAA | 60 | 282 | 279-294 | 16 | Panel 3 |
| *De novo* | - | Nuclear | (GA)_24_ | TAAATACAACCTCTCTCGCTCTAG | AGGCATTGTGGCAGAAAATC | 58 | 330 | 279-334 | 42 | Panel 3 |
| *De novo* | - | Nuclear | (TC)_15_ | ATCTAAGGCACCCGTTCTCT | GAAAATATCTCTGCGTGCG | 58 | 303 | 290-321 | 20 | Panel 3 |
| *De novo* | - | Nuclear | (AT)_10_ | CTTTTACGCCTTGGCTATCAA | CAGTGTCCATCTCTAGTTCAAAATG | 59 | 167 | 155-197 | 37 | Panel 3 |
| *De novo* | - | Chloroplast | (AT)_5_ | GATTCACAAGTCCAATCCCAAT | TGGCTGGAAAAGTTCTATTGCT | 60 | 235 | 233-235 | 3 | Panel 3 |
| *De novo* | - | Mitochondria | (AT)_6_ | TATAGTGGGGTTGAAACACGAG | GACCGTTCGCTTTCTCAGTAG | 59 | 299 | 298-316 | 4 | Panel 3 |
| *De novo* | - | Nuclear | (TC)_17_ | TCGAAAAGGATGATGAGGATCT | ACCTGAACCAACAACGACAAC | 60 | 243 | 229-245 | 11 | Panel 4 |
| *De novo* | - | Nuclear | (AC)_15_ | CTCATTCACTATTGTACTTGTTGGC | ATGGAAAAGAAACCGCACA | 59 | 153 | 136-165 | 20 | Panel 4 |
| *De novo* | - | Chloroplast | (AT)_5_ | CATGGATGAGGTACTAGATGGTGA | AACCAACCCAAACACAAAACTC | 60 | 383 | Poor | 2 | Panel 4 |
| *De novo* | - | Chloroplast | (T)_13_ | CTTTGGTGAGATCCAAGAGTTTC | TTATATGTCCACCTTGTTCCCC | 60 | 308 | Poor | 3 | Panel 4 |
| *De novo* | - | Chloroplast | (TA)_9_ | ATTCTGCGTACCATATCCCAAA | GGCCCCTTGTAACTTCTAACAAA | 60 | 201 | Poor | 2 | Panel 4 |
| *De novo* | - | Chloroplast | (T)_10_ | AGCTTTATCATCCTGGTGTTGAG | AGAAGTTTGAGCCTTTGTTTGC | 60 | 332 | Poor | 3 | Panel 4 |

**Table S2.** Mean raw and retained and percentage aligned RAD-seq reads per population in this study. Population are ordered by Latitude. Three individuals failed (AS031, BE012, BW022) and revised sample sizes are given in parenthesis.

| Population | ID | Seq Lane | Samples | Mean raw reads | Mean retained reads | Percentage aligned |
| --- | --- | --- | --- | --- | --- | --- |
| Nordkapp | NK | 356 | 6 | 1,543,810 | 1,285,795 | 86.14 |
| Sirbma | SM | 356 | 6 | 1,481,561 | 1,238,025 | 86.31 |
| Tenontie | TE | 356 | 6 | 1,055,522 | 891,215 | 84.53 |
| Skalluvaara | SK | 356 | 6 | 1,326,412 | 1,125,210 | 86.82 |
| Kevo Plateau | KP | 356 | 6 | 1,709,576 | 1,450,323 | 87.44 |
| Kevojarvi | KA | 356 | 6 | 1,422,712 | 1,190,226 | 87.45 |
| Gearddosjavri | UG | 356 | 6 | 2,021,033 | 1,706,841 | 87.20 |
| Kevo Reserve | KR | 356 | 6 | 1,938,219 | 1,585,083 | 86.25 |
| Kotilampi | DJ | 356 | 6 | 1,679,537 | 1,419,872 | 87.56 |
| Partakko | PT | 356 | 6 | 1,635,143 | 1,384,462 | 89.02 |
| Ben Loyal | BL | 356 | 6 | 1,382,713 | 1,141,459 | 61.11 |
| Meall Odhar | MO | 355 | 6 | 1,218,902 | 1,054,081 | 74.83 |
| Beinn Enaiglair | BE | 355 | 5(6) | 1,699,026 | 1,384,636 | 62.86 |
| Luichart | LH | 355 | 6 | 1,495,995 | 1,300,669 | 75.01 |
| Ben Wyvis W | BW | 356 | 5(6) | 1,480,757 | 1,219,949 | 70.68 |
| Loch Meig | ME | 355 | 6 | 1,566,948 | 1,359,440 | 82.87 |
| Glen Cannich | GC | 355/356 | 6 | 1,099,895 | 936,821 | 88.60 |
| Dundreggan | DE | 356 | 6 | 1,344,040 | 1,112,968 | 78.01 |
| An Suidhe | AS | 355 | 2(3) | 1,290,769 | 1,127,370 | 87.16 |
| Beinn Bhreac | BB | 356 | 6 | 2,237,543 | 1,815,379 | 47.10 |
| Portclair | PC | 355 | 6 | 1,500,806 | 1,327,653 | 80.87 |
| River Avon | AV | 356 | 6 | 2,066,494 | 1,723,052 | 78.20 |
| Monadhliaths | MD | 355 | 6 | 2,130,440 | 1,860,040 | 68.45 |
| Meall an t'slugain | SL | 355 | 6 | 1,735,207 | 1,509,525 | 69.42 |
| Loch Muick E | MU1 | 355 | 6 | 1,907,437 | 1,677,637 | 65.29 |
| Loch Muick W | MU2 | 355 | 6 | 2,075,747 | 1,819,636 | 78.73 |
| Loch Laggan | LG | 355 | 6 | 1,311,284 | 1,137,807 | 87.00 |
| Loch Loch | LL | 355 | 6 | 1,873,293 | 1,660,542 | 75.03 |
| Ben Gullabin | BG | 355 | 1 | 1,470,190 | 1,330,773 | 82.73 |
| Loch Rannoch | LR | 355 | 6 | 1,521,827 | 1,354,679 | 82.96 |
| Rannoch West | RW | 355 | 6 | 1,694,284 | 1,500,466 | 85.85 |
| Rannoch Moor B | RB | 355 | 6 | 1,537,317 | 1,335,699 | 87.61 |
| Lennox | LX | 355 | 2 | 2,537,407 | 2,107,081 | 43.07 |
| Emblehope | EM | 355 | 1 | 2,275,265 | 1,936,689 | 67.25 |
| Spadeadam | SA | 355 | 1 | 1,804,816 | 1,632,967 | 84.56 |
| Teesdale | TD | 355 | 2 | 2,057,576 | 1,727,514 | 41.41 |

**Table S3.** Prior parameters used in DIY-ABC analysis. Population size units are number of individuals. Time units are in generations**.**

| Variable | Description | Scenario 1v2 |  | Scenario 1v1b |  |
| --- | --- | --- | --- | --- | --- |
|  |  | Lower | Upper | Lower | Upper |
| AV | Current effective population size | - | - | 10 | 60 |
| BB | Current effective population size | - | - | 10 | 50 |
| BL | Current effective population size | - | - | 10 | 80 |
| BW | Current effective population size | - | - | 20 | 200 |
| GC | Current effective population size | - | - | 10 | 60 |
| LG | Current effective population size | - | - | 10 | 45 |
| LL | Current effective population size | - | - | 10 | 40 |
| MU2 | Current effective population size | - | - | 50 | 2000 |
| RW | Current effective population size | - | - | 20 | 1000 |
| NeSc | Current effective population size | 10 | 10000 | 3000 | 12000 |
| NeBr | Current effective meta-population size | 10 | 10000 | 100 | 4000 |
| NeH | Historic effective population size | 10 | 10000 | 3000 | 12000 |
| tb1 | Putative contemporary bottleneck | 10 | 3000 | 10 | 1000 |
| tb2 | Putative historic bottleneck | 10 | 3000 | - | - |
| t1 | Divergence of populations | 10 | 3000 | 10 | 1000 |
| t2 | Divergence of Scandinavian and British lineages | 10 | 3000 | 10 | 1400 |
| Defined priors: t2>t1, t1>tb1. | | | |  |  |

**Table S4.** Microsatellite prior parameters used in DIY-ABC analysis.

| Marker | Parameter | Distribution | Minimum | Maximum | Mean |
| --- | --- | --- | --- | --- | --- |
| PCR-SSR | Mean mutation rate (u) | uniform | 1.00E-04 | 4.00E-03 | 0.0005 |
|  | Individual locus mutation rate | gamma | 1.00E-05 | 1.00E-02 | Mean u |
|  | Mean coefficient (P) | uniform | 1.00E-01 | 4.00E-01 | 0.22 |
|  | Individual locus coefficient | gamma | 1.00E-02 | 9.00E-01 | Mean P |
| RAD-SSR | Mean mutation rate (u) | uniform | 1.00E-04 | 1.00E-03 | 0.0005 |
|  | Individual locus mutation rate | gamma | 1.00E-05 | 1.00E-02 | Mean u |
|  | Mean coefficient (P) | uniform | 1.00E-01 | 3.00E-01 | 0.22 |
|  | Individual locus coefficient | gamma | 1.00E-02 | 9.00E-01 | Mean P |

**Table S5.** Summary statistics used in DIY-ABC analysis.

| Summary Statistic | PCR-SSR | RAD-SSR | RAD-SNP_ti_ | RAD-SNP_tv_ |
| --- | --- | --- | --- | --- |
| **Individual based** |  |  |  |  |
| Mean number of alleles | x | x |  |  |
| Mean genic diversity | x | x | x | x |
| Variance of genic diversity |  |  | x | x |
| Mean size variance | x |  |  |  |
| Mean Garza-Williamson's M |  |  |  |  |
| **Population based** |  |  |  |  |
| Mean number of alleles | x | x |  |  |
| Mean genic diversity | x | x |  |  |
| Mean size variance | x |  |  |  |
| F_st_ | x | x | x | x |
| Variance F_st_ |  |  | x | x |
| Shared allele distance |  | x |  |  |

**Table S6.** Summary statistics for all populations ordered by decreasing latitude. Allelic richness (A_R_) is rarefied to two diploid individual. Means are reported for Scandinavia, large British and small British populations.

| **Pop** | **A_R_** | | | | **P** | | | | **H_O_** | | | | **H_E_** | | | | **F_IS_** | | | |
| --- | --- | --- | --- | --- | --- | --- | --- | --- | --- | --- | --- | --- | --- | --- | --- | --- | --- | --- | --- | --- |
|  | PCR-SSR | RAD-SSR | RAD-SNP_ti_ | RAD-SNP_tv_ | PCR-SSR | RAD-SSR | RAD-SNP_ti_ | RAD-SNP_tv_ | PCR-SSR | RAD-SSR | RAD-SNP_ti_ | RAD-SNP_tv_ | PCR-SSR | RAD-SSR | RAD-SNP_ti_ | RAD-SNP_tv_ | PCR-SSR | RAD-SSR | RAD-SNP_ti_ | RAD-SNP_tv_ |
| **NK** | 1.722 | 1.389 | 1.111 | 1.105 | 0.132 | 0.096 | 0.012 | 0.010 | 0.448 | 0.188 | 0.043 | 0.043 | 0.591 | 0.301 | 0.098 | 0.099 | 0.264 | 0.288 | 0.156 | 0.150 |
| **SM** | 1.737 | 1.518 | 1.120 | 1.109 | 0.183 | 0.180 | 0.012 | 0.009 | 0.503 | 0.172 | 0.047 | 0.046 | 0.603 | 0.292 | 0.105 | 0.106 | 0.194 | 0.314 | 0.156 | 0.161 |
| **TE** | 1.716 | 1.454 | 1.108 | 1.104 | 0.129 | 0.127 | 0.016 | 0.014 | 0.557 | 0.158 | 0.033 | 0.034 | 0.702 | 0.246 | 0.092 | 0.096 | 0.211 | 0.305 | 0.152 | 0.156 |
| **SK** | 1.687 | 1.448 | 1.114 | 1.103 | 0.223 | 0.159 | 0.011 | 0.012 | 0.481 | 0.188 | 0.040 | 0.041 | 0.552 | 0.290 | 0.100 | 0.102 | 0.144 | 0.308 | 0.163 | 0.154 |
| **KP** | 1.696 | 1.532 | 1.117 | 1.112 | 0.160 | 0.154 | 0.013 | 0.015 | 0.454 | 0.229 | 0.051 | 0.050 | 0.569 | 0.327 | 0.105 | 0.106 | 0.202 | 0.293 | 0.151 | 0.145 |
| **KA** | 1.666 | 1.377 | 1.104 | 1.100 | 0.099 | 0.115 | 0.009 | 0.011 | 0.451 | 0.189 | 0.044 | 0.044 | 0.545 | 0.261 | 0.092 | 0.095 | 0.167 | 0.242 | 0.136 | 0.131 |
| **UG** | 1.755 | 1.494 | 1.113 | 1.108 | 0.292 | 0.178 | 0.013 | 0.008 | 0.487 | 0.264 | 0.056 | 0.056 | 0.604 | 0.341 | 0.101 | 0.100 | 0.224 | 0.218 | 0.127 | 0.125 |
| **KR** | 1.705 | 1.419 | 1.116 | 1.109 | 0.142 | 0.162 | 0.011 | 0.013 | 0.452 | 0.218 | 0.052 | 0.052 | 0.577 | 0.318 | 0.103 | 0.105 | 0.240 | 0.263 | 0.147 | 0.137 |
| **DJ** | 1.683 | 1.431 | 1.117 | 1.108 | 0.138 | 0.156 | 0.012 | 0.010 | 0.461 | 0.233 | 0.053 | 0.054 | 0.559 | 0.316 | 0.104 | 0.106 | 0.220 | 0.243 | 0.148 | 0.146 |
| **PT** | 1.727 | 1.423 | 1.116 | 1.114 | 0.155 | 0.117 | 0.011 | 0.009 | 0.497 | 0.231 | 0.049 | 0.050 | 0.592 | 0.317 | 0.103 | 0.105 | 0.193 | 0.258 | 0.150 | 0.159 |
| **Scan. mean** | 1.710 | 1.448 | 1.114 | 1.107 | 0.165 | 0.144 | 0.012 | 0.011 | 0.479 | 0.207 | 0.047 | 0.047 | 0.589 | 0.301 | 0.100 | 0.102 | 0.206 | 0.273 | 0.148 | 0.146 |
| **BL** | 1.676 | 1.470 | 1.091 | 1.086 | 0.104 | 0.169 | 0.005 | 0.009 | 0.529 | 0.136 | 0.029 | 0.030 | 0.662 | 0.220 | 0.075 | 0.074 | 0.202 | 0.325 | 0.113 | 0.121 |
| **MO** | 1.659 | 1.340 | 1.102 | 1.097 | 0.052 | 0.067 | 0.007 | 0.010 | 0.604 | 0.083 | 0.030 | 0.029 | 0.645 | 0.197 | 0.085 | 0.085 | 0.067 | 0.298 | 0.137 | 0.140 |
| **BE** | 1.699 | 1.355 | 1.083 | 1.083 | 0.156 | 0.075 | 0.004 | 0.007 | 0.562 | 0.082 | 0.028 | 0.028 | 0.684 | 0.166 | 0.065 | 0.066 | 0.179 | 0.274 | 0.107 | 0.101 |
| **LH** | 1.685 | 1.351 | 1.106 | 1.092 | 0.099 | 0.087 | 0.008 | 0.009 | 0.578 | 0.081 | 0.034 | 0.034 | 0.672 | 0.211 | 0.090 | 0.092 | 0.142 | 0.322 | 0.155 | 0.126 |
| **BW** | 1.714 | 1.435 | 1.094 | 1.084 | 0.092 | 0.143 | 0.007 | 0.007 | 0.575 | 0.144 | 0.035 | 0.036 | 0.700 | 0.206 | 0.075 | 0.075 | 0.196 | 0.250 | 0.101 | 0.108 |
| **DG** | 1.686 | - | - | - | 0.095 | - | - | - | 0.572 |  | - | - | 0.667 |  | - | - | 0.179 | - | - | - |
| **ME** | 1.724 | 1.348 | 1.102 | 1.103 | 0.131 | 0.094 | 0.008 | 0.008 | 0.563 | 0.091 | 0.037 | 0.037 | 0.697 | 0.207 | 0.088 | 0.090 | 0.218 | 0.298 | 0.140 | 0.150 |
| **GC** | 1.679 | 1.372 | 1.102 | 1.106 | 0.065 | 0.086 | 0.007 | 0.007 | 0.553 | 0.161 | 0.032 | 0.033 | 0.665 | 0.221 | 0.086 | 0.087 | 0.181 | 0.257 | 0.141 | 0.147 |
| **FS** | 1.669 | - | - | - | 0.112 | - | - | - | 0.394 |  | - | - | 0.545 |  | - | - | 0.259 | - | - | - |
| **DE** | 1.734 | 1.431 | 1.105 | 1.101 | 0.160 | 0.127 | 0.006 | 0.008 | 0.552 | 0.172 | 0.033 | 0.033 | 0.718 | 0.265 | 0.090 | 0.091 | 0.246 | 0.294 | 0.153 | 0.143 |
| **AS** | 1.699 | 1.233 | 1.060 | 1.057 | 0.117 | 0.081 | 0.005 | 0.008 | 0.528 | 0.083 | 0.036 | 0.035 | 0.684 | 0.084 | 0.034 | 0.032 | 0.255 | 0.113 | 0.029 | 0.031 |
| **BB** | 1.695 | 1.494 | 1.096 | 1.101 | 0.102 | 0.222 | 0.008 | 0.007 | 0.541 | 0.183 | 0.035 | 0.035 | 0.682 | 0.284 | 0.082 | 0.080 | 0.244 | 0.277 | 0.121 | 0.119 |
| **PC** | 1.680 | 1.372 | 1.099 | 1.097 | 0.098 | 0.081 | 0.009 | 0.009 | 0.538 | 0.124 | 0.040 | 0.040 | 0.670 | 0.239 | 0.085 | 0.085 | 0.199 | 0.270 | 0.122 | 0.123 |
| **AV** | 1.646 | 1.492 | 1.098 | 1.086 | 0.109 | 0.146 | 0.006 | 0.006 | 0.526 | 0.259 | 0.051 | 0.053 | 0.633 | 0.309 | 0.086 | 0.089 | 0.169 | 0.240 | 0.105 | 0.095 |
| **MD** | 1.659 | 1.435 | 1.100 | 1.092 | 0.107 | 0.063 | 0.011 | 0.005 | 0.571 | 0.131 | 0.040 | 0.040 | 0.649 | 0.269 | 0.088 | 0.089 | 0.161 | 0.336 | 0.125 | 0.108 |
| **SL** | 1.695 | 1.415 | 1.100 | 1.093 | 0.058 | 0.047 | 0.007 | 0.006 | 0.605 | 0.107 | 0.038 | 0.038 | 0.683 | 0.227 | 0.086 | 0.085 | 0.147 | 0.350 | 0.125 | 0.125 |
| **MU1** | 1.602 | 1.354 | 1.096 | 1.090 | 0.108 | 0.062 | 0.007 | 0.004 | 0.515 | 0.097 | 0.034 | 0.034 | 0.591 | 0.173 | 0.083 | 0.085 | 0.162 | 0.270 | 0.130 | 0.123 |
| **MU2** | 1.569 | 1.327 | 1.103 | 1.087 | 0.076 | 0.055 | 0.005 | 0.004 | 0.465 | 0.106 | 0.041 | 0.042 | 0.559 | 0.222 | 0.091 | 0.091 | 0.168 | 0.303 | 0.128 | 0.117 |
| **LG** | 1.646 | 1.364 | 1.095 | 1.093 | 0.051 | 0.074 | 0.008 | 0.009 | 0.570 | 0.101 | 0.031 | 0.031 | 0.639 | 0.197 | 0.080 | 0.079 | 0.110 | 0.288 | 0.125 | 0.133 |
| **LL** | 1.651 | 1.455 | 1.104 | 1.096 | 0.061 | 0.080 | 0.005 | 0.008 | 0.497 | 0.131 | 0.044 | 0.044 | 0.637 | 0.246 | 0.091 | 0.089 | 0.234 | 0.323 | 0.123 | 0.122 |
| **LR** | 1.665 | 1.330 | 1.099 | 1.095 | 0.110 | 0.121 | 0.008 | 0.005 | 0.616 | 0.106 | 0.040 | 0.040 | 0.649 | 0.200 | 0.086 | 0.087 | 0.077 | 0.255 | 0.122 | 0.113 |
| **RW** | 1.670 | 1.392 | 1.102 | 1.097 | 0.090 | 0.088 | 0.008 | 0.005 | 0.547 | 0.136 | 0.039 | 0.039 | 0.659 | 0.248 | 0.090 | 0.089 | 0.169 | 0.265 | 0.140 | 0.124 |
| **RB** | 1.630 | 1.310 | 1.094 | 1.091 | 0.091 | 0.055 | 0.008 | 0.007 | 0.580 | 0.102 | 0.040 | 0.041 | 0.619 | 0.226 | 0.082 | 0.083 | 0.087 | 0.266 | 0.112 | 0.123 |
| **RA** | 1.645 | - | - | - | 0.107 | - | - | - | 0.568 |  | - | - | 0.633 |  | - | - | 0.118 | - | - | - |
| **Brit. Mean (large)** | 1.670 | 1.384 | 1.097 | 1.092 | 0.098 | 0.096 | 0.007 | 0.007 | 0.548 | 0.125 | 0.036 | 0.037 | 0.652 | 0.220 | 0.082 | 0.082 | 0.174 | 0.280 | 0.122 | 0.119 |
| **BG** | 1.431 | 1.200 | 1.032 | 1.038 | 0.031 | 0.110 | 0.011 | 0.009 | 0.478 | 0.078 | 0.023 | 0.022 | 0.386 | 0.039 | 0.011 | 0.011 | 0.002 | - | - |  |
| **LX** | 1.566 | 1.233 | 1.048 | 1.057 | 0.075 | 0.079 | 0.006 | 0.005 | 0.354 | 0.060 | 0.019 | 0.019 | 0.442 | 0.057 | 0.028 | 0.028 | 0.244 | 0.063 | 0.033 | 0.052 |
| **EM** | 1.583 | 1.300 | 1.029 | 1.040 | 0.169 | 0.108 | 0.005 | 0.007 | 0.561 | 0.078 | 0.022 | 0.025 | 0.426 | 0.039 | 0.011 | 0.012 | 0.067 | 0.000 | 0.000 |  |
| **SA** | 1.389 | 1.300 | 1.042 | 1.036 | 0.063 | 0.055 | 0.008 | 0.002 | 0.369 | 0.109 | 0.031 | 0.031 | 0.212 | 0.054 | 0.016 | 0.015 | 0.000 | 0.000 | 0.000 |  |
| **TD** | 1.667 | 1.167 | 1.038 | 1.045 | 0.119 | 0.110 | 0.005 | 0.005 | 0.667 | 0.039 | 0.013 | 0.013 | 0.500 | 0.047 | 0.016 | 0.015 | -0.006 | 0.093 | 0.024 | 0.032 |
| **Brit. Mean (all)** | 1.527 | 1.240 | 1.038 | 1.043 | 0.092 | 0.092 | 0.007 | 0.006 | 0.486 | 0.073 | 0.022 | 0.022 | 0.393 | 0.047 | 0.016 | 0.016 | 0.061 | 0.039 | 0.014 | 0.042 |

| **Table S7.** Population selfing rate estimates based on PCR-SSR markers, for dwarf birch populations in the UK and Scandinavia. Mean estimates are generated overall for each region. We have excluded estimates from populations with small sample sizes, as they lack precision. | | | | | |
| --- | --- | --- | --- | --- | --- |
| Population | g_2_ method | | | ML method | |
|  | s | S.D. | p-value | ML estimate | CI (0.05-0.95) |
| NK^1^ | 0.17 | 0.09 | 0.01 | 0.14 | (0-0.25) |
| SM | 0 | 0 | 0.67 | 0.08 | (0-0.22) |
| TE | 0 | 0 | 0.98 | 0 | (0-0.08) |
| SK | 0 | 0 | 0.59 | 0 | (0-0.14) |
| KP | 0 | 0 | 0.51 | 0 | (0-0.1) |
| KA | 0.03 | 0.07 | 0.35 | 0 | (0-0.08) |
| UG | 0.02 | 0.10 | 0.41 | 0.02 | (0-0.28) |
| KR | 0.01 | 0.07 | 0.45 | 0.04 | (0-0.17) |
| DJ | 0 | 0 | 0.54 | 0 | (0-0.13) |
| PT | 0 | 0 | 0.56 | 0 | (0-0.14) |
| Scan. mean | 0.02 |  |  | 0.03 | (0-0.04) |
| BL | 0.11 | 0.08 | 0.07 | 0 | (0-0.1) |
| MO | 0.00 | 0.08 | 0.47 | 0 | (0-0.09) |
| BE | 0 | 0 | 0.79 | 0 | (0-0.12) |
| LH | 0.12 | 0.08 | 0.04 | 0 | (0-0.11) |
| BW | 0.06 | 0.07 | 0.22 | 0 | (0-0.17) |
| DG | 0 | 0 | 0.79 | 0 | (0-0.1) |
| ME | 0.05 | 0.08 | 0.25 | 0.04 | (0-0.17) |
| GC | 0 | 0 | 0.49 | 0.05 | (0-0.22) |
| FS | 0.13 | 0.08 | 0.02 | 0.14 | (0.03-0.24) |
| DE | 0 | 0 | 0.99 | 0 | (0-0.11) |
| AS | 0 | 0 | 0.65 | 0 | (0-0.05) |
| BB | 0.06 | 0.07 | 0.18 | 0 | (0-0.08) |
| PC | 0.03 | 0.06 | 0.27 | 0 | (0-0.09) |
| AV | 0.10 | 0.08 | 0.05 | 0 | (0-0.2) |
| MD | 0 | 0 | 0.77 | 0 | (0-0.07) |
| SL | 0 | 0 | 0.52 | 0.07 | (0-0.2) |
| MU1 | 0 | 0 | 0.52 | 0 | (0-0.05) |
| MU2 | 0.05 | 0.06 | 0.13 | 0.03 | (0-0.16) |
| LG | 0 | 0 | 0.89 | 0 | (0-0.07) |
| LL | 0.01 | 0.05 | 0.42 | 0.07 | (0.01-0.14) |
| LR | 0.26 | 0.09 | 0.01 | 0 | (0-0.17) |
| RW | 0.03 | 0.07 | 0.30 | 0.03 | (0-0.15) |
| RA | 0 | 0 | 0.68 | 0 | (0-0.08) |
| RB | 0.05 | 0.07 | 0.22 | 0 | (0-0.1) |
| Brit. mean (large) | 0.04 |  |  | 0.02 | (0-0.02) |

^1^ Scandinavian selfing estimates are upwards biased due to a high value at population NK. This is a relatively small and isolated population, close to the Northern limit of the *Betula nana* distribution. Therefore a comparatively high selfing estimate for this population is not unexpected.

**Table S8.** Maximum likelihood F_ST_ across all markers and PCR-SSR based effective population size (N_e_) and census population estimates for all populations.

| **Pop** | **Maximum likelihood F_ST_ (95% CI)** | | | | **N_e_ (95% CI)** | **Census** |
| --- | --- | --- | --- | --- | --- | --- |
|  | PCR-SSR | RAD-SSR | RAD-SNP_ti_ | RAD-SNP_tv_ | PCR-SSR |  |
| **NK** | 0.01 (0.00-0.01) | 0.06 (0.04-0.08) | 0.08 (0.08-0.09) | 0.10 (0.09-0.11) | 313.9 (104.3-∞) | 10-100 |
| **SM** | 0.00 (0.00-0.01) | 0.05 (0.03-0.07) | 0.09 (0.08-0.10) | 0.09 (0.08-0.10) | 1915.7 (113.2-∞) | 100-1000 |
| **TE** | 0.01 (0.00-0.02) | 0.11 (0.07-0.15) | 0.14 (0.13-0.15) | 0.13 (0.12-0.15) | -159.9 (2583.5-∞)^1^ | 100-1000 |
| **SK** | 0.02 (0.01-0.03) | 0.05 (0.03-0.08) | 0.08 (0.08-0.09) | 0.09 (0.08-0.11) | 227.8 (37.9-∞) | 100-1000 |
| **KP** | 0.01 (0.00-0.01) | 0.04 (0.03-0.06) | 0.06 (0.06-0.07) | 0.06 (0.06-0.07) | 218.1 (77.6-∞) | 100-1000 |
| **KA** | 0.01 (0.01-0.02) | 0.07 (0.05-0.1) | 0.14 (0.13-0.15) | 0.14 (0.13-0.16) | 68.1 (42.8-143.8) | 100-1000 |
| **UG** | 0.02 (0.01-0.04) | 0.03 (0.02-0.05) | 0.09 (0.08-0.09) | 0.09 (0.09-0.10) | 112.4 (22.3-∞) | 1000-10000 |
| **KR** | 0.00 (0.00-0.01) | 0.02 (0.01-0.04) | 0.07 (0.06-0.07) | 0.07 (0.06-0.08) | 12446.5 (132.4-∞) | 1000-10000 |
| **DJ** | 0.01 (0.01-0.02) | 0.05 (0.04-0.08) | 0.06 (0.06-0.07) | 0.08 (0.07-0.09) | 93.7 (45-1525.6) | 100-1000 |
| **PT** | 0.00 (0.00-0.01) | 0.06 (0.04-0.09) | 0.07 (0.07-0.08) | 0.06 (0.06-0.07) | 227.6 (99.8-∞) | 1000-10000 |
| **Scan. mean** | 0.009 | 0.054 | 0.088 | 0.091 | 1546.4 | - |
| **BL** | 0.06 (0.04-0.08) | 0.12 (0.09-0.17) | 0.23 (0.22-0.25) | 0.22 (0.20-0.24) | 30.5 (22.3-45.1) | 10-100 |
| **MO** | 0.08 (0.05-0.11) | 0.16 (0.11-0.22) | 0.18 (0.17-0.20) | 0.15 (0.13-0.16) | 11.4 (8.9-14.7) | 25 |
| **BE** | 0.04 (0.03-0.06) | 0.28 (0.21-0.36) | 0.24 (0.22-0.26) | 0.22 (0.20-0.25) | -428.9 (216-∞)^1^ | 10-100 |
| **LH** | 0.03 (0.02-0.04) | 0.19 (0.14-0.25) | 0.15 (0.14-0.16) | 0.19 (0.17-0.21) | 49.4 (35.7-75.1) | 10-100 |
| **BW** | 0.02 (0.01-0.03) | 0.15 (0.1-0.2) | 0.22 (0.20-0.24) | 0.21 (0.18-0.23) | 99.2 (52.8-447.8) | 100-1000 |
| **DG** | 0.02 (0.01-0.03) | - | - | - | 36.1 (25.1-59.1) | 10-100 |
| **ME** | 0.03 (0.02-0.04) | 0.21 (0.16-0.27) | 0.18 (0.17-0.19) | 0.16 (0.14-0.17) | 74.2 (37-559.9) | 10-100 |
| **GC** | 0.04 (0.03-0.06) | 0.13 (0.1-0.18) | 0.20 (0.18-0.21) | 0.16 (0.14-0.17) | 52.8 (34.4-100.3) | 66 |
| **FS** | 0.04 (0.03-0.06) | - | - | - | 744.8 (93.7-∞) | 100-1000 |
| **DE** | 0.02 (0.02-0.03) | 0.04 (0.02-0.06) | 0.13 (0.12-0.14) | 0.11 (0.10-0.12) | 44.3 (28.6-85.9) | 38 |
| **AS** | 0.02 (0.01-0.03) | 0.41 (0.29-0.53) | 0.41 (0.37-0.45) | 0.39 (0.34-0.44) | 85.7 (43.6-528.9) | 10-100 |
| **BB** | 0.03 (0.02-0.05) | 0.07 (0.05-0.1) | 0.20 (0.18-0.21) | 0.16 (0.15-0.18) | 30.6 (23.6-41.4) | 50 |
| **PC** | 0.05 (0.04-0.07) | 0.17 (0.13-0.22) | 0.19 (0.18-0.21) | 0.18 (0.16-0.19) | 14.8 (12.4-17.8) | 41 |
| **AV** | 0.10 (0.08-0.14) | 0.04 (0.03-0.06) | 0.15 (0.14-0.16) | 0.17 (0.16-0.19) | 62.1 (35.6-172) | 60 |
| **MD** | 0.09 (0.07-0.12) | 0.17 (0.14-0.22) | 0.19 (0.18-0.20) | 0.21 (0.19-0.22) | 10.8 (9.1-12.7) | 10-100 |
| **SL** | 0.03 (0.02-0.05) | 0.14 (0.1-0.18) | 0.20 (0.19-0.21) | 0.18 (0.16-0.19) | 273.2 (95.5-∞) | 10-100 |
| **MU1** | 0.10 (0.07-0.13) | 0.24 (0.19-0.31) | 0.23 (0.22-0.25) | 0.20 (0.19-0.22) | 591 (85.9-∞) | 1000-10000 |
| **MU2** | 0.12 (0.09-0.16) | 0.22 (0.18-0.28) | 0.19 (0.18-0.20) | 0.20 (0.18-0.22) | 714.9 (95.8-∞) | 1000-10000 |
| **LG** | 0.05 (0.04-0.07) | 0.26 (0.2-0.31) | 0.23 (0.22-0.25) | 0.20 (0.18-0.21) | 15.1 (12.6-18.1) | 49 |
| **LL** | 0.11 (0.08-0.15) | 0.13 (0.1-0.17) | 0.19 (0.18-0.20) | 0.20 (0.18-0.21) | 13.4 (10.4-17.4) | 10-100 |
| **LR** | 0.10 (0.08-0.14) | 0.24 (0.19-0.3) | 0.19 (0.17-0.20) | 0.19 (0.17-0.21) | 6 (4.4-7.6) | 29 |
| **RW** | 0.06 (0.04-0.09) | 0.16 (0.13-0.2) | 0.16 (0.15-0.17) | 0.13 (0.12-0.15) | 32.4 (23.8-47.1) | 1000-10000 |
| **RA** | 0.09 (0.06-0.12) | - | - | - | 13.7 (10.3-18.4) | 100-1000 |
| **RB** | 0.08 (0.06-0.10) | 0.19 (0.15-0.24) | 0.25 (0.23-0.26) | 0.23 (0.22-0.25) | 55.3 (37.3-97.3) | 100-1000 |
| **Brit. mean (large)** | 0.058 | 0.177 | 0.205 | 0.193 | 109.7 | - |
| **BG** | 0.42 (0.29-0.57) | 0.68 (0.52-0.8) | 0.77 (0.73-0.81) | 0.67 (0.61-0.73) | 0.5 (0.4-0.6) | 5 |
| **LX** | 0.19 (0.12-0.30) | 0.51 (0.37-0.65) | 0.53 (0.49-0.57) | 0.45 (0.40-0.50) | 1.2 (1-1.5) | 9 |
| **EM** | 0.42 (0.29-0.56) | 0.64 (0.47-0.78) | 0.72 (0.67-0.76) | 0.65 (0.59-0.71) | 0.7 (0.5-1) | 2 |
| **SA** | 0.68 (0.51-0.83) | 0.54 (0.39-0.69) | 0.67 (0.62-0.72) | 0.70 (0.64-0.75) | -1.6 (∞-∞) | 1 |
| **TD** | 0.03 (0.00-0.13) | 0.5 (0.34-0.65) | 0.62 (0.57-0.67) | 0.58 (0.52-0.63) | -1 (∞-∞) | 2 |
| **Brit. mean (all)** | 0.108 | 0.253 | 0.293 | 0.273 | 90.8 | - |

^1^ Ngative estimates are indicative of insufficient sample size, with the size correction being greater than the *r*ˆ2 value calculated for the data. Two populations with poor N_e_ estimates were excluded from Figures 3 and 4.

**Table S9.** Bias and precision of posterior estimates across marker sets using DIY-ABC.

|  | | | |  | | | | Posterior distribution | | | | Posterior median | | | |
| --- | --- | --- | --- | --- | --- | --- | --- | --- | --- | --- | --- | --- | --- | --- | --- |
|  | Param. | | True value | | RRMISE | | RMAD | | 50% cov. | | 95% cov. | | MRB | | fact2 |
| *PCR-SSR* | |  | |  | |  | |  | |  | |  | |  | |
|  | AV | | 41.7 | | 0.236 | | 0.178 | | 0.652 | | 0.986 | | 0.001 | | 1.00 |
|  | BB | | 48.7 | | 0.133 | | 0.092 | | 0.178 | | 0.736 | | -0.081 | | 1.00 |
|  | BL | | 51.5 | | 0.278 | | 0.21 | | 0.586 | | 0.974 | | 0.068 | | 1.00 |
|  | BW | | 118.5 | | 0.339 | | 0.256 | | 0.544 | | 0.952 | | 0.029 | | 1.00 |
|  | GC | | 52.8 | | 0.155 | | 0.11 | | 0.466 | | 0.952 | | -0.051 | | 1.00 |
|  | LG | | 43.3 | | 0.155 | | 0.109 | | 0.148 | | 0.696 | | -0.095 | | 1.00 |
|  | LL | | 30.0 | | 0.217 | | 0.162 | | 0.616 | | 0.982 | | 0.001 | | 1.00 |
|  | MU2 | | 27.9 | | 1.965 | | 0.393 | | 0.554 | | 0.944 | | 0.262 | | 1.00 |
|  | RW | | 56.0 | | 1.428 | | 0.53 | | 0.706 | | 0.992 | | 0.254 | | 0.994 |
|  | NeSc | | 6640 | | 0.320 | | 0.248 | | 0.516 | | 0.976 | | -0.019 | | 0.986 |
|  | NeBr | | 7456 | | 0.341 | | 0.261 | | 0.51 | | 0.954 | | -0.060 | | 0.966 |
|  | tb1 | | 10.0 | | 0.031 | | 0.009 | | 0.978 | | 1.00 | | 0.004 | | 1.00 |
|  | t1 | | 98.4 | | 6.198 | | 2.88 | | 0.36 | | 0.862 | | 1.018 | | 0.448 |
|  | t2 | | 975.0 | | 0.375 | | 0.279 | | 0.46 | | 0.948 | | 0.033 | | 0.97 |
|  | µmic_1 | | 8.90E-04 | | 0.822 | | 0.564 | | 0.456 | | 0.942 | | 0.222 | | 0.922 |
|  | pmic_1 | | 2.21E-01 | | 0.543 | | 0.396 | | 0.574 | | 0.96 | | 0.085 | | 0.888 |
|  | snimic_1 | | 3.04E-06 | | 24.075 | | 4.784 | | 0.454 | | 0.924 | | -0.228 | | 0.11 |
| *RAD-SSR* | |  | |  | |  | |  | |  | |  | |  | |
|  | AV | | 51.9 | | 0.245 | | 0.177 | | 0.398 | | 0.912 | | -0.093 | | 0.998 |
|  | BB | | 42.4 | | 0.262 | | 0.193 | | 0.396 | | 0.912 | | -0.079 | | 0.992 |
|  | BL | | 33.6 | | 0.679 | | 0.479 | | 0.494 | | 0.948 | | 0.235 | | 0.952 |
|  | BW | | 32.9 | | 1.136 | | 0.640 | | 0.464 | | 0.908 | | 0.427 | | 0.926 |
|  | GC | | 40.0 | | 0.400 | | 0.290 | | 0.484 | | 0.954 | | 0.049 | | 0.98 |
|  | LG | | 29.6 | | 0.394 | | 0.288 | | 0.488 | | 0.966 | | 0.054 | | 0.998 |
|  | LL | | 36.3 | | 0.217 | | 0.154 | | 0.366 | | 0.912 | | -0.090 | | 1.00 |
|  | MU2 | | 64.4 | | 4.744 | | 2.021 | | 0.160 | | 0.520 | | 1.553 | | 0.744 |
|  | RW | | 61.8 | | 2.726 | | 1.217 | | 0.576 | | 0.954 | | 0.801 | | 0.85 |
|  | NeSc | | 7970 | | 0.287 | | 0.231 | | 0.430 | | 0.922 | | -0.136 | | 1.00 |
|  | NeBr | | 6060 | | 0.449 | | 0.340 | | 0.456 | | 0.932 | | 0.179 | | 0.998 |
|  | tb1 | | 10.6 | | 0.167 | | 0.106 | | 0.362 | | 0.954 | | 0.041 | | 1.00 |
|  | t1 | | 396 | | 2.266 | | 0.947 | | 0.548 | | 0.988 | | 0.055 | | 0.778 |
|  | t2 | | 805 | | 0.427 | | 0.319 | | 0.550 | | 0.966 | | 0.070 | | 0.998 |
|  | µmic_1 | | 1.33E-04 | | 0.536 | | 0.373 | | 0.346 | | 0.836 | | 0.273 | | 0.992 |
|  | pmic_1 | | 2.09E-01 | | 0.375 | | 0.297 | | 0.506 | | 0.946 | | -0.101 | | 0.998 |
|  | snimic_1 | | 9.70E-07 | | 61.47 | | 17.28 | | 0.520 | | 0.954 | | 3.479 | | 0.252 |
| *RAD-SNP_t_*_i_ | |  | |  | |  | |  | |  | |  | |  | |
|  | AV | | 55.2 | | 0.128 | | 0.095 | | 0.416 | | 0.946 | | -0.056 | | 1.00 |
|  | BB | | 38.5 | | 0.204 | | 0.155 | | 0.452 | | 0.960 | | 0.055 | | 1.00 |
|  | BL | | 37.3 | | 0.439 | | 0.316 | | 0.584 | | 0.972 | | 0.170 | | 1.00 |
|  | BW | | 25.6 | | 0.461 | | 0.209 | | 0.532 | | 0.964 | | 0.074 | | 1.00 |
|  | GC | | 38.0 | | 0.296 | | 0.222 | | 0.582 | | 0.974 | | 0.089 | | 1.00 |
|  | LG | | 30.0 | | 0.266 | | 0.204 | | 0.586 | | 0.976 | | 0.061 | | 1.00 |
|  | LL | | 38.6 | | 0.107 | | 0.071 | | 0.324 | | 0.972 | | -0.046 | | 1.00 |
|  | MU2 | | 50.4 | | 1.491 | | 0.305 | | 0.036 | | 0.384 | | 0.250 | | 0.998 |
|  | RW | | 45.2 | | 1.610 | | 0.554 | | 0.676 | | 0.996 | | 0.271 | | 0.992 |
|  | NeSc | | 9070 | | 0.244 | | 0.194 | | 0.254 | | 0.866 | | -0.163 | | 1.00 |
|  | NeBr | | 6120 | | 0.388 | | 0.293 | | 0.554 | | 0.964 | | 0.125 | | 1.00 |
|  | tb1 | | 11.5 | | 0.143 | | 0.103 | | 0.502 | | 0.952 | | -0.021 | | 1.00 |
|  | t1 | | 21.1 | | 12.97 | | 8.212 | | 0.058 | | 0.462 | | 7.535 | | 0.10 |
|  | t2 | | 998 | | 0.258 | | 0.207 | | 0.564 | | 0.976 | | -0.084 | | 1.00 |
| *RAD-SNP_tv_* | |  | |  | |  | |  | |  | |  | |  | |
|  | AV | | 43.5 | | 0.235 | | 0.181 | | 0.572 | | 0.972 | | 0.005 | | 1.00 |
|  | BB | | 42.1 | | 0.191 | | 0.145 | | 0.494 | | 0.944 | | -0.04 | | 1.00 |
|  | BL | | 37.1 | | 0.422 | | 0.308 | | 0.596 | | 0.972 | | 0.083 | | 0.998 |
|  | BW | | 41.2 | | 0.649 | | 0.365 | | 0.474 | | 0.966 | | 0.091 | | 0.984 |
|  | GC | | 43.4 | | 0.249 | | 0.191 | | 0.556 | | 0.964 | | 0.00 | | 1.00 |
|  | LG | | 38.8 | | 0.189 | | 0.144 | | 0.468 | | 0.942 | | -0.054 | | 1.00 |
|  | LL | | 37.2 | | 0.166 | | 0.122 | | 0.342 | | 0.894 | | -0.079 | | 1.00 |
|  | MU2 | | 50.4 | | 1.374 | | 0.276 | | 0.128 | | 0.612 | | 0.272 | | 0.998 |
|  | RW | | 46.9 | | 1.413 | | 0.496 | | 0.486 | | 0.972 | | 0.209 | | 0.992 |
|  | NeSc | | 8150 | | 0.268 | | 0.210 | | 0.422 | | 0.950 | | -0.044 | | 1.00 |
|  | NeBr | | 6450 | | 0.401 | | 0.306 | | 0.484 | | 0.954 | | 0.108 | | 1.00 |
|  | tb1 | | 15.1 | | 0.224 | | 0.180 | | 0.330 | | 0.912 | | -0.112 | | 1.00 |
|  | t1 | | 108 | | 7.254 | | 4.141 | | 0.280 | | 0.864 | | 3.934 | | 0.328 |
|  | t2 | | 914 | | 0.322 | | 0.247 | | 0.582 | | 0.964 | | 0.015 | | 1.00 |

## Supplementary Figures


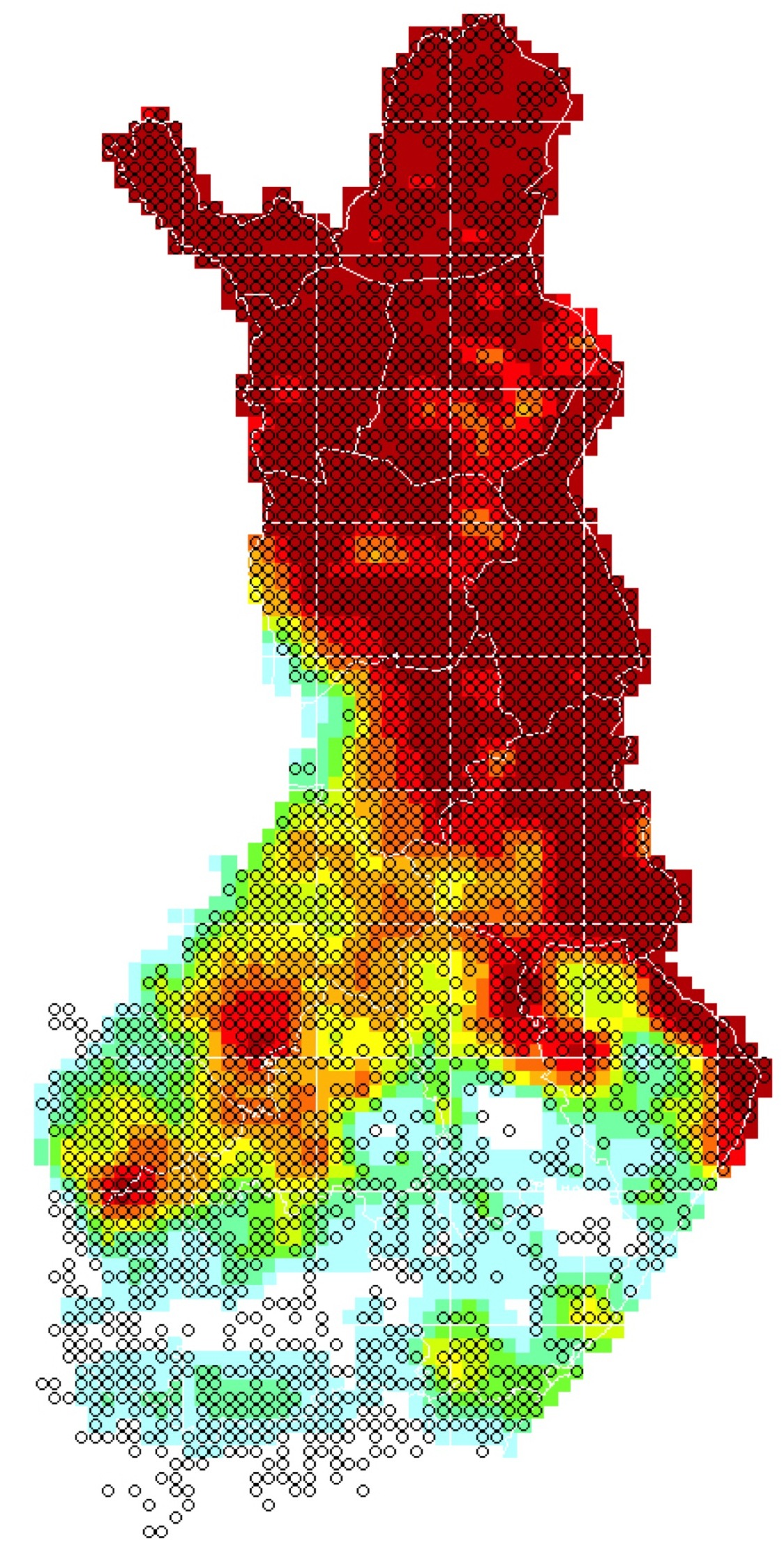

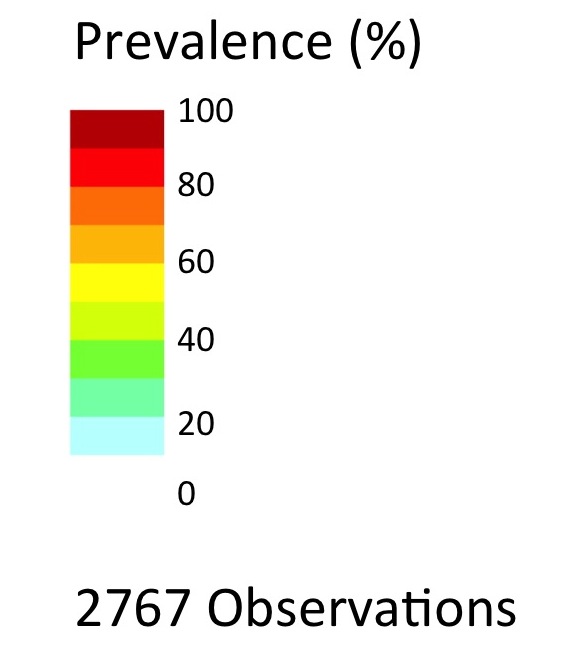


**Figure S1.** Prevalence of *B. nana* in Finland with rectangle denoting main study area. Figure is adapted from Lampinen, R. Lahti, T. 2016: Plant Atlas 2015 - University of Helsinki, Museum of Natural History, Helsinki. ([www.luomus.fi/kasviatlas/](http://www.luomus.fi/kasviatlas/))


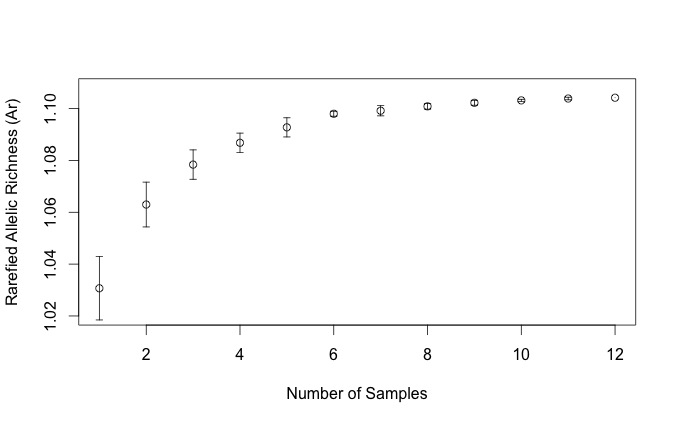
**Figure S2.** Response of rarefied allelic richness (A_r_) estimates to increasing sample size in a combined Loch Muick (MU1 and MU2) population.

**Figure S3.** Population based Expected heterozygosity plotted against latitude for all four marker types.


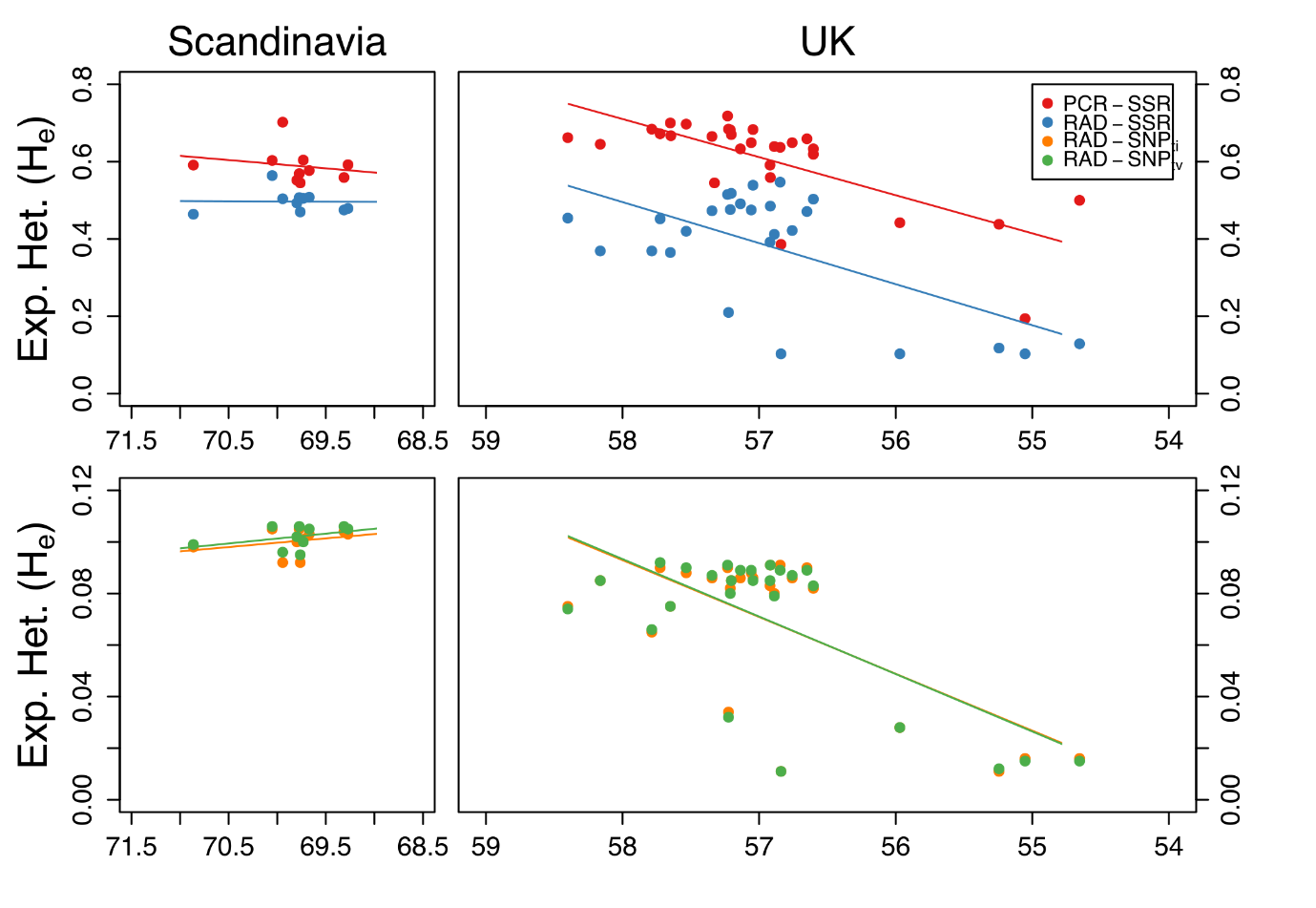


Scandinavia

Britain

**A)**


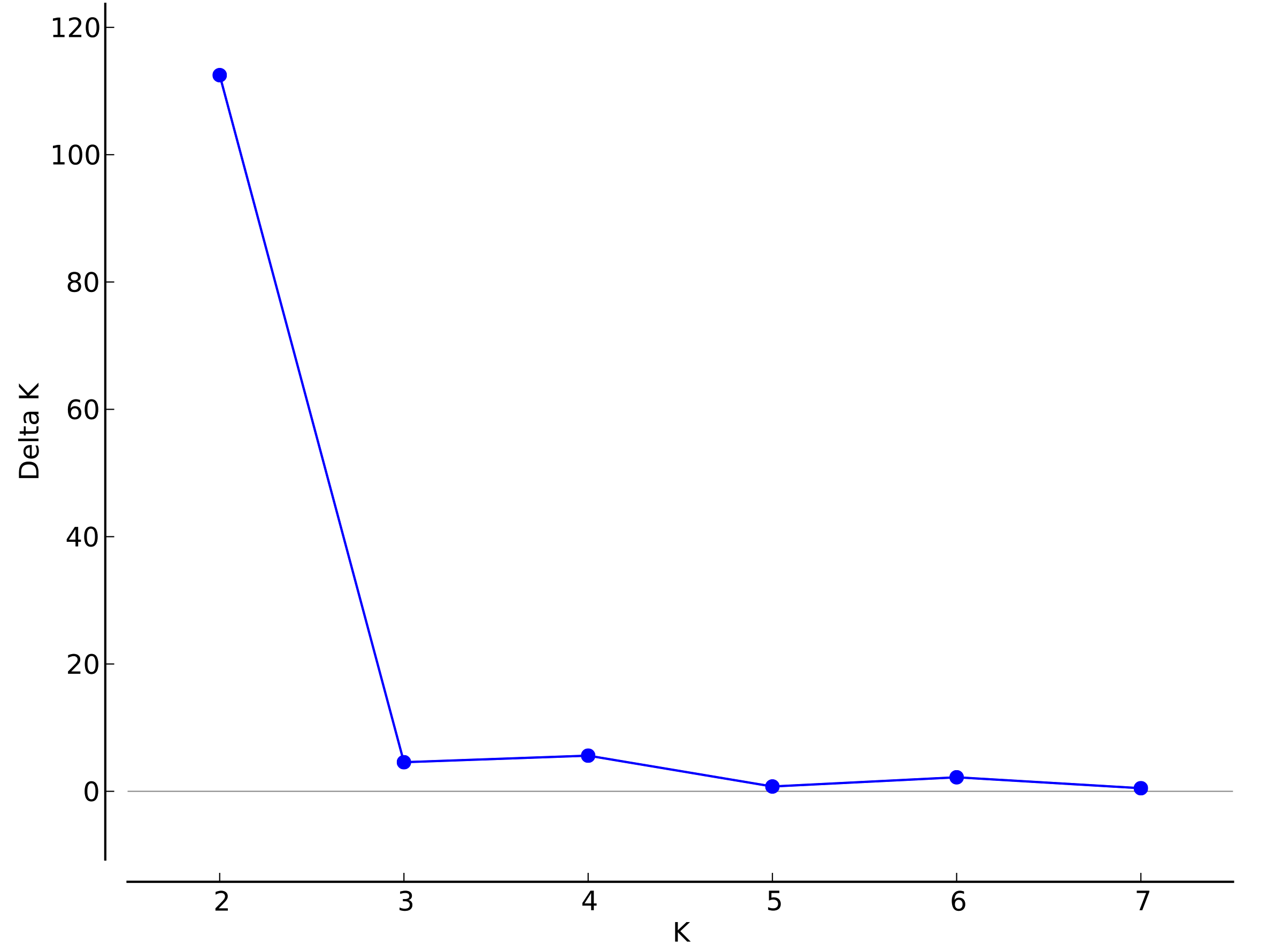


**B)**


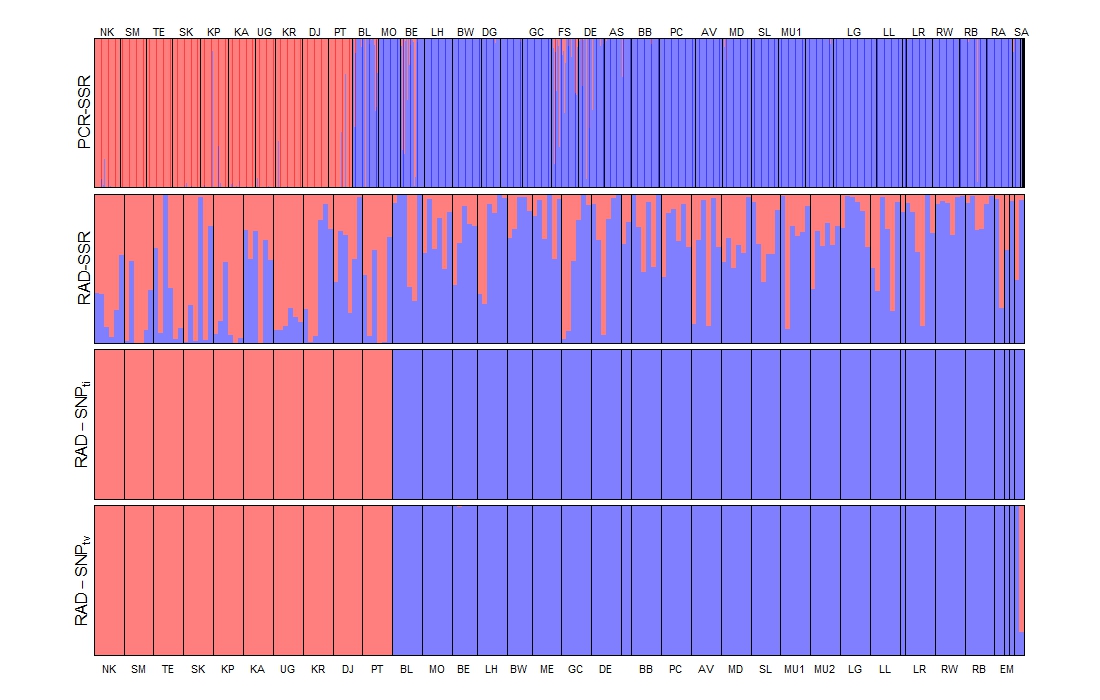


**Figure S4.** A) Ln P(D) plot indicating likelihood support for different values of K, for marker set PCR-SSR. B) STRUCTURE plots of *Betula nana* for all marker sets with individuals ordered from left to right by decreasing latitude. The Evanno DeltaK method clearly identifies k=2 for all datasets. The PCR-SSR dataset consists of 1066 individuals (population IDs above the plot), and the other three datasets consist of 187 individuals (population IDs below the plot).


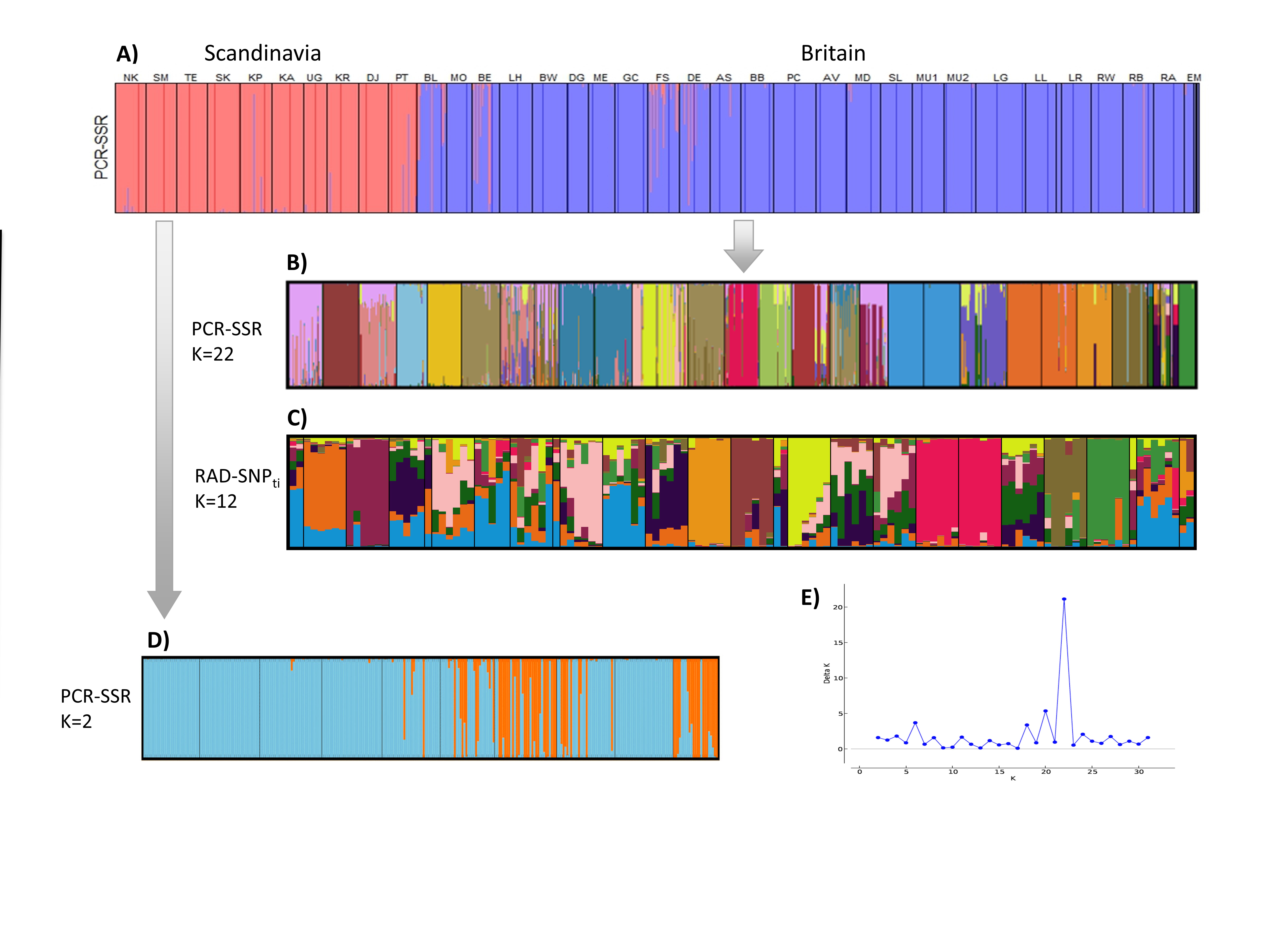
**Figure S5.** A) STRUCTURE plot of *Betula nana* for all Scandinavian and British samples, based on PCR-SSR markers as shown in Figure S4. B) STRUCTURE plot of British populations based on PCR-SSR markers, where the best supported value is k=22 (see inset plot E). C) STRUCTURE plots of *Betula nana* for RAD-SNP_ti_ markers, where the best supported value is K=12. D) STRUCTURE plot of Scandinavian populations illustrating little clear population structure with a best supported value of k=2.


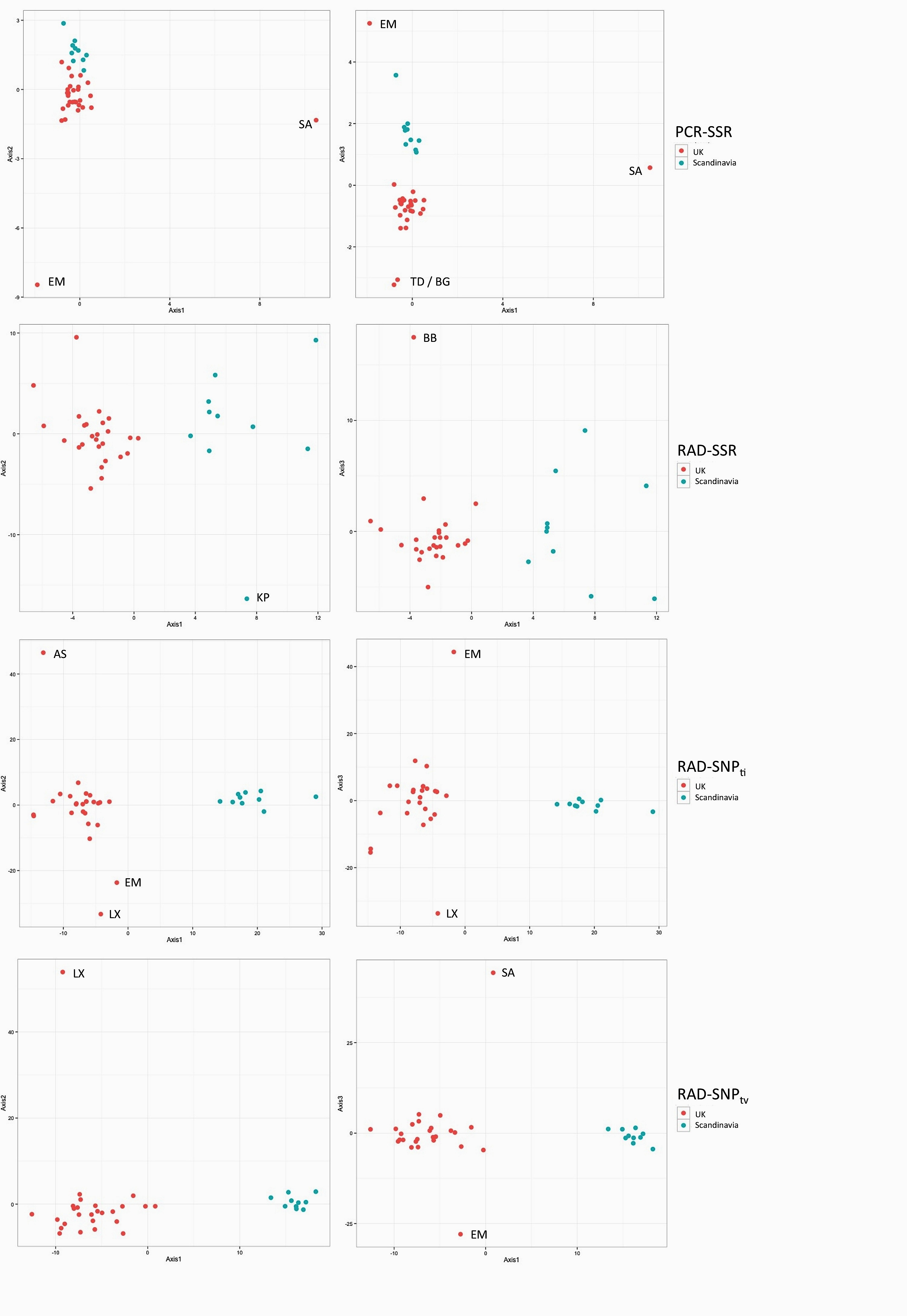


**Figure S6.** Principal component analysis of all four genetic marker datasets colored by geographic region.


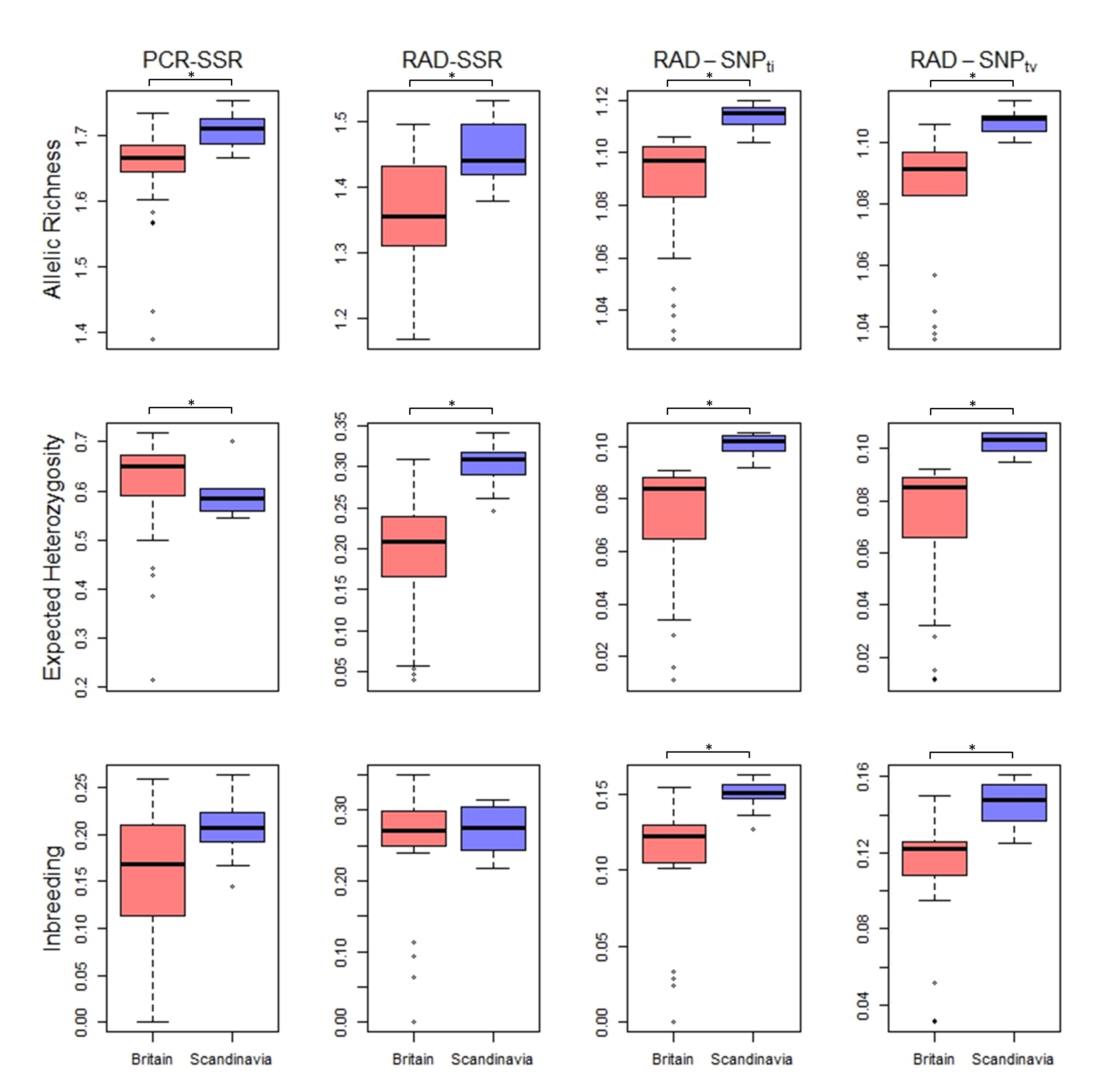


**Figure S7.** Boxplots of population based genetic statistics grouped by marker and region (see Table S6 for raw data). Asterisk denotes significance in T-tests (p≤0.01).


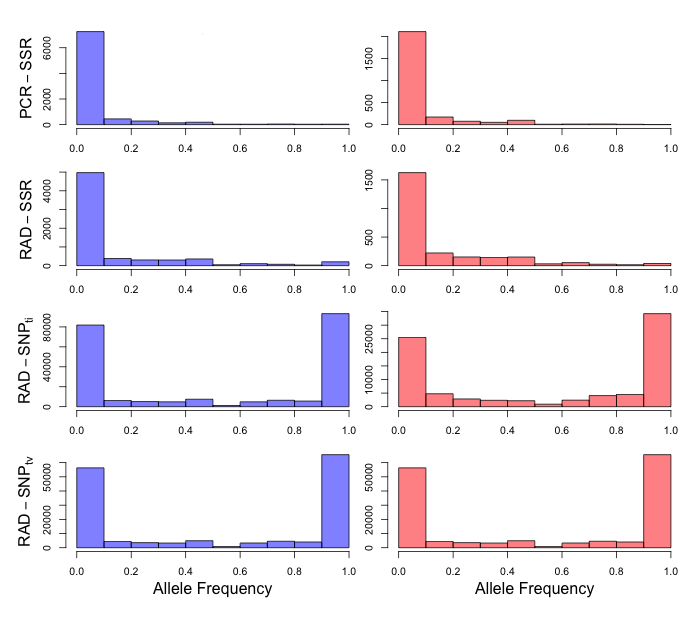


Britain

Scandinavia

**Figure S8.** Allele frequency spectra across marker types, pooled by study regions. The x-axis represents categories for the major allele with frequency on the y-axis. The majority of variable loci are at very low or high frequencies indicating they are fixed/absent in many populations.

**
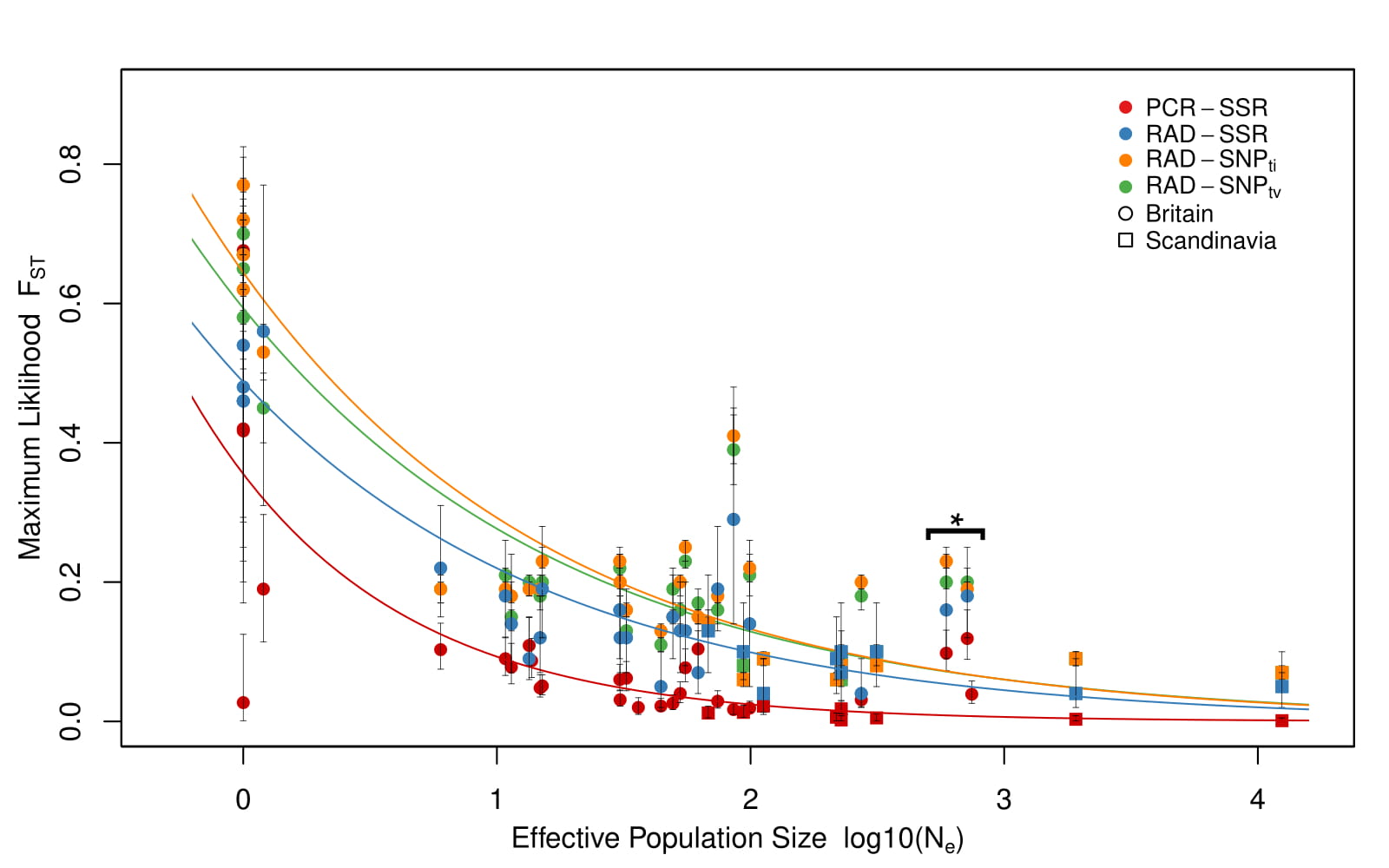
Figure S9.** Scatter plot of the relationship between linkage disequilibrium based log effective population size (N_e_) and maximum likelihood F_ST_, for each marker type, using all datasets across both regions. Populations MU1 and MU2, denoted by ‘*’ are outliers with a large effective population size, but also moderate differentiation consistent across markers.


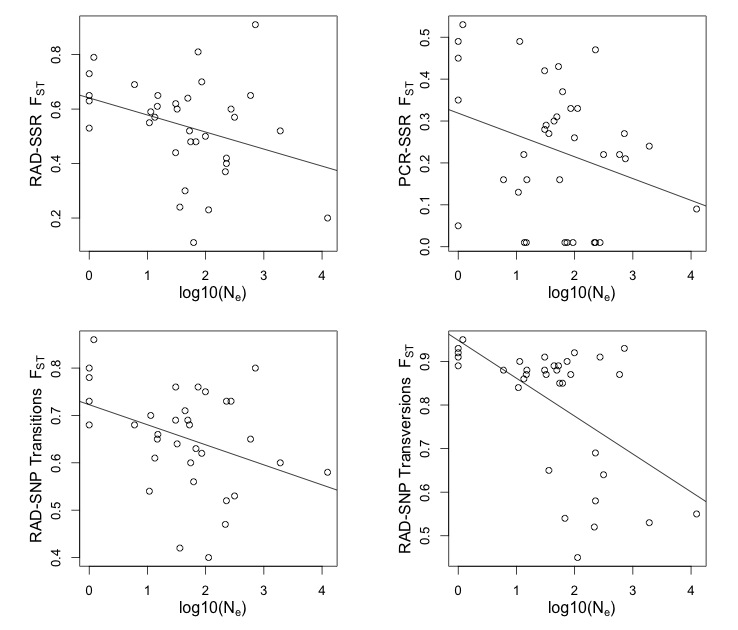


**Figure S10.** Plots of effective population size and maximum likelihood F_ST_, for a randomly selected single individual from each *Betula nana* study population, across all datasets and both regions. Linear regression was significant (p<0.05) for all RAD- datasets and p=0.068 for the PCR-SSR dataset.


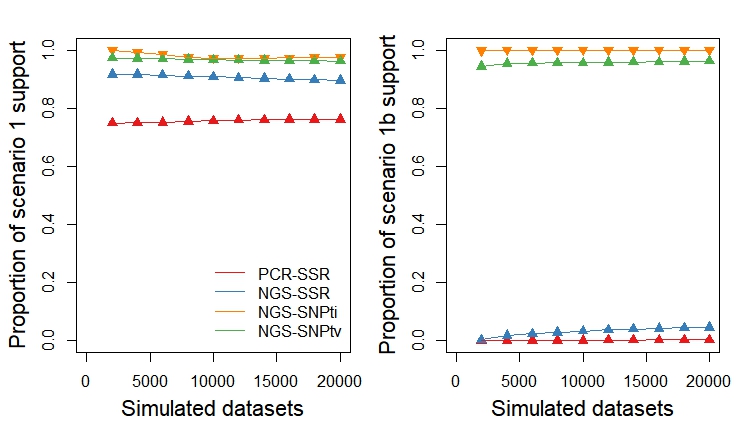


**Figure S11.** Logistic regression support for scenario 1 (in comparison sc1 v sc2) and scenario 1b (in comparison sc1 v sc1b) in DIY-ABC.


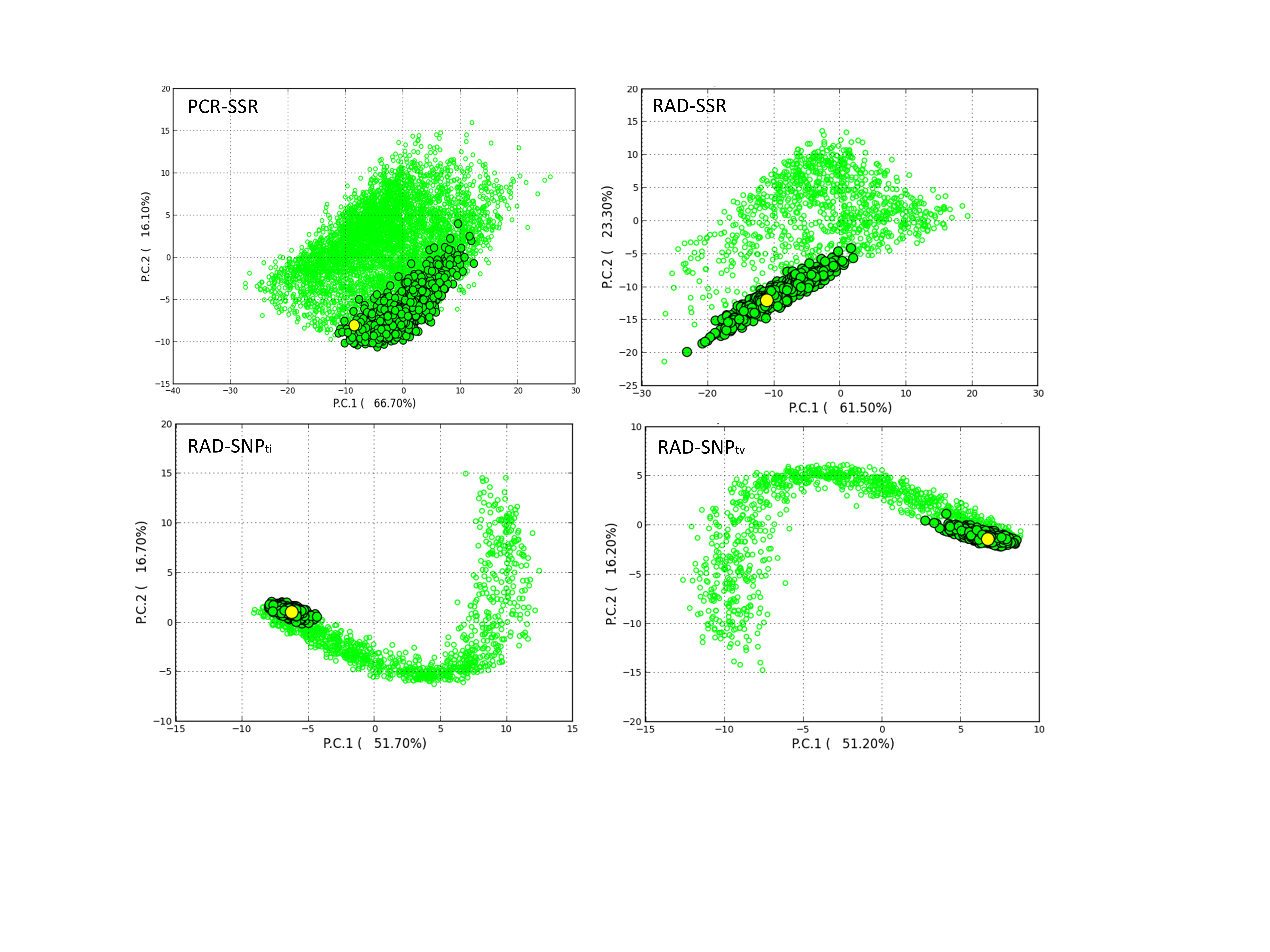


**Figure S12.** Model checking evaluation across all markers for scenario 1b.

## Supplementary References

Benestan, L. M. *et al.* (2016) ‘Conservation genomics of natural and managed populations: Building a conceptual and practical framework’, *Molecular Ecology*, pp. 2967–2977. doi: 10.1111/mec.13647.

Chaplin, F. S. I. (1980) ‘Nutrient Allocation and Responses to Defoliation in Tundra Plants’, *Arctic and Alpine Research*, 12(4), pp. 553–563.

David, P. *et al.* (2007) ‘Reliable selfing rate estimates from imperfect population genetic data’, *Molecular Ecology*, 16(12), pp. 2474–2487. doi: 10.1111/j.1365-294X.2007.03330.x.

Earl, D. A. and vonHoldt, B. M. (2012) ‘STRUCTURE HARVESTER: A website and program for visualizing STRUCTURE output and implementing the Evanno method’, *Conservation Genetics Resources*, 4(2), pp. 359–361. doi: 10.1007/s12686-011-9548-7.

Evanno, G., Regnaut, S. and Goudet, J. (2005) ‘Detecting the number of clusters of individuals using the software STRUCTURE: A simulation study’, *Molecular Ecology*, 14(8), pp. 2611–2620. doi: 10.1111/j.1365-294X.2005.02553.x.

Falush, D., Dorp, L. van and Lawson, D. (2016) ‘A tutorial on how (not) to over-interpret STRUCTURE/ADMIXTURE bar plots’, *bioRxiv*, p. 066431. doi: 10.1101/066431.

Hubisz, M. J. *et al.* (2009) ‘Inferring weak population structure with the assistance of sample group information’, *Molecular Ecology Resources*, 9(5), pp. 1322–1332. doi: 10.1111/j.1755-0998.2009.02591.x.

Jombart, T. (2008) ‘Adegenet: A R package for the multivariate analysis of genetic markers’, *Bioinformatics*, 24(11), pp. 1403–1405. doi: 10.1093/bioinformatics/btn129.

Kopelman, N. M. *et al.* (2015) ‘Clumpak: A program for identifying clustering modes and packaging population structure inferences across K’, *Molecular Ecology Resources*, 15(5), pp. 1179–1191. doi: 10.1111/1755-0998.12387.

Pritchard, J. K., Stephens, M. and Donnelly, P. (2000) ‘Inference of population structure using multilocus genotype data’, *Genetics*, 155(2), pp. 945–959. doi: 10.1111/j.1471-8286.2007.01758.x.

R Development Core Team (2014) ‘R: A language and environment for statistical computing’, *Foundation for Statistical Computing, Vienna, Austria.*

## Appendix 1: Maximum Likelihood F_ST_ Script

Maximum Liklihood F_ST_ and RAD-SSR genotyping functions are available at <https://github.com/orgs/SBCSnicholsLab/***>

##########################################################################

# Function to return log( P(?? | p,Fst) ), the log multinomial dirichlet calculation of the likelihood of

# allelic composition ??,

# given Fst f,

# and allele frequencies p

# n.b. this log version deals well with small values of f and p

# Warning: p values must be non-zero

##########################################################################

lmd_test<-function(a=c(2,8),f=0.1,p=c(0.3,0.7)){ #n.b. these default values illustrate a simple call

l<-1/f-1;n=sum(a);x<-l*p

return(lgamma(l)+lgamma(n+1)-lgamma(n+l)

+sum(lgamma(x+a))

-sum(lgamma(a+1))

-sum(lgamma(x))

)

}

lmd <- function(a,f,p) {

l<-1/f-1;n=sum(a);x<-l*p

return(lgamma(l)+lgamma(n+1)-lgamma(n+l)

+sum(lgamma(x+a))

-sum(lgamma(a+1))

-sum(lgamma(x))

)

}

# Vectorize the function so it can act over a vector different f values

lmdL<-Vectorize(lmd,'f')

##########################################################################

##########################################################################

# Function to return sum(log( P(?? | p,Fst) )), for

# al: a list of allelic counts for different loci (??)

# ap: a list of allele frequencies (p)

# f: a vector of Fst values (f)

# Warning p-values should be non-zero

##########################################################################

combinedL<-function(al,pl,f){

if (length(al) != length(pl)) {

stop('The number of loci in the population is different than in the super-population')

}

L <- rep(0,length(f))

for (i in 1:length(al)){

L <- L+lmdL(al[[i]],f,pl[[i]])

}

return(L)

}

##########################################################################

# Main Block

##########################################################################

# Example data

Locus1Counts<-c(1,4,30)

Locus2Counts<-c(20,3,2,7)

Locus3Counts<-c(30,2)

xalist<-list(Locus1Counts,Locus2Counts,Locus3Counts)

Locus1p<-c(.3,.6,.1)

Locus2p<-c(.2,.2,.2,.4)

Locus3p<-c(.5,.5)

xplist<-list(Locus1p,Locus2p,Locus3p)

# Ploting likelihood curve

xvals<-1:999/1000

Lcurve<-combinedL(xalist,f=xvals,xplist)

plot(xvals,Lcurve,type='l')

# Get ML value

peakV<-max(Lcurve,na.rm=T)

MLv<-xvals[which(Lcurve==peakV)]

# Get support limits

inside<-which(Lcurve>=(peakV-2))

supportLims<-c(xvals[inside][1],tail(xvals[inside],1))

abline(v=supportLims,col='red')

lines(supportLims,rep(peakV-2,2),col='red')

##########################################################################
